# Functional Reconstitution of a Voltage-Gated Potassium Channel in Giant Unilamellar Vesicles

**DOI:** 10.1371/journal.pone.0025529

**Published:** 2011-10-06

**Authors:** Sophie Aimon, John Manzi, Daniel Schmidt, Jose Antonio Poveda Larrosa, Patricia Bassereau, Gilman E. S. Toombes

**Affiliations:** 1 Unité Mixte de Recherche (UMR) 168, Physico-Chimie Curie, Centre National de la Recherche Scientifique (CNRS), Institut Curie, Centre de Recherche, Université Pierre et Marie Curie, Paris, France; 2 Howard Hughes Medical Institute, Laboratory of Molecular Neurobiology and Biophysics, Rockefeller University, New York, New York, United States of America; 3 Instituto de Biología Molecular y Celular, Universidad Miguel Hernández, Elche, Spain; Swiss Federal Institute of Technology Zurich, Switzerland

## Abstract

Voltage-gated ion channels are key players in cellular excitability. Recent studies suggest that their behavior can depend strongly on the membrane lipid composition and physical state. *In vivo* studies of membrane/channel and channel/channel interactions are challenging as membrane properties are actively regulated in living cells, and are difficult to control in experimental settings. We developed a method to reconstitute functional voltage-gated ion channels into cell-sized Giant Unilamellar Vesicles (GUVs) in which membrane composition, tension and geometry can be controlled. First, a voltage-gated potassium channel, KvAP, was purified, fluorescently labeled and reconstituted into small proteoliposomes. Small proteoliposomes were then converted into GUVs via electroformation. GUVs could be formed using different lipid compositions and buffers containing low (5 mM) or near-physiological (100 mM) salt concentrations. Protein incorporation into GUVs was characterized with quantitative confocal microscopy, and the protein density of GUVs was comparable to the small proteoliposomes from which they were formed. Furthermore, patch-clamp measurements confirmed that the reconstituted channels retained potassium selectivity and voltage-gated activation. GUVs containing functional voltage-gated ion channels will allow the study of channel activity, distribution and diffusion while controlling membrane state, and should prove a powerful tool for understanding how the membrane modulates cellular excitability.

## Introduction

Voltage-gated potassium (Kv) channels are a family of transmembrane proteins which open and close potassium-selective pores in response to changes in cell membrane potential [1]. The flow of potassium ions through Kv channels modulates cellular excitability and they play a crucial role in many processes such as neural signaling and muscle contraction. Many diseases are caused by mutations that alter Kv channel kinetics [2], [3], [4], and the precise localization of specific Kv channel types on the axon and dendrites is required for proper signaling in neurons [5]. Consequently, Kv channels have been intensively studied to understand the channel structure, physics of channel gating and mechanisms controlling channel localization [6].

Functional, biochemical and theoretical studies suggest that the surrounding membrane can influence Kv channel function and localization. For example, Kv2.1 channels were activated in membranes in which sphingomyelin was converted to ceramide-1-phosphate [7], while the lipid, phosphatidylinositol-4,5-bisphosphate (PIP2), was reported to activate Kv7 channels [8]. In addition to specific protein-lipid interactions, the physical state of the membrane (charge, tension, thickness, curvature, etc.) may influence channel function [9], [10]. Indeed, it has been suggested that small peptide toxins targeting voltage-dependent ion channels act by altering the forces exerted by the membrane on the channel [11]. The membrane may also influence channel localization through physical mechanisms involving heterogeneities in membrane composition, mechanical properties and shape. Biochemical and functional data [12] have been used to infer the localization of several Kv channel types in so-called “lipid rafts”, dynamic small membrane domains enriched in cholesterol and sphingomyelin [13]. Membrane curvature could also influence channel distribution [14], while membrane thickness has been predicted to affect channel clustering [15]. There is thus great interest in understanding the interactions between Kv channels and the membrane.

Studying the effects of membrane properties on channel function and localization *in vivo* is challenging as the composition and physical state of the membrane is highly regulated by the cell itself. Consequently, much work has been done to isolate membrane proteins and reconstitute them in membranes of controlled composition. For electrophysiological studies of ion channel activity, several *in vitro* systems have been developed including black lipid membranes (BLMs) and patch-clamp on multilamellar vesicles [16]. However, each technique has distinct limitations concerning the control of membrane tension, shape or composition. BLMs, for example, often contain residual solvent and the membrane tension cannot be readily controlled [17], [18].

**Figure 1:**
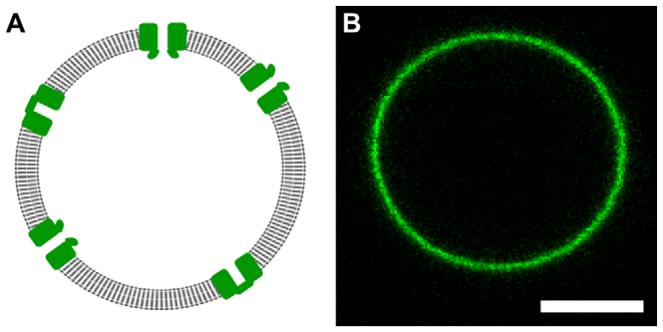
Giant unilamellar vesicle containing reconstituted KvAP. A) Cartoon of ion channels (green) inserted into the single lipid bilayer (gray; thickness ∼5 nm) of a GUV (diameter ∼10 µm; not at scale). Many techniques used to study cell membranes can be applied to GUVs because they are unilamellar and of comparable size. B) Confocal image of a representative GUV containing KvAP labeled with Alexa488. Scale bar: 5 µm.

Giant Unilamellar Vesicles (GUVs) are a promising alternative *in vitro* system. These cell-sized unilamellar liposomes (Figure 1) can be prepared from a wide range of lipid compositions while both membrane tension and curvature can be controlled. Micropipette aspiration can be used to set GUV membrane tension over a wide, and biologically relevant, range (∼10^−7^ N/m to 10^−3^ N/m) [19]. Although the GUV membrane is effectively flat on the molecular scale, a curved membrane can be formed by extracting a cylindrical membrane tube (“tether”) from the GUV [20]. Competition between the membrane bending energy and tension determines the tube radius, R, which can be controlled over a biologically relevant range of membrane curvatures (R∼10 nm to 200 nm) [21]. The protein distribution in the GUV and membrane nanotube can be monitored with fluorescence microscopy, while channel activity can be measured with electrophysiology techniques. Thus, GUVs offer the possibility to study channel activity and distribution while controlling membrane composition, tension and curvature.

Realizing the potential of this system requires efficient methods to incorporate functional Kv channels into GUVs. Classically, GUVs have been prepared using a lipid film deposited from organic solvent, a procedure likely to damage all but the toughest proteins [22]. Thus, one difficulty is to find conditions that preserve protein activity while still allowing a high yield of GUVs. To date, efforts have been made to reconstitute several ion channels into GUVs including the bacterial mechanosensitive channel MscL [23], several porins [24], [25], [26], an unidentified chloride channel [27], the potassium channel, KcsA [28], and Connexin-26 [29]. Still, the functional incorporation of complex membrane proteins into GUVs remains challenging [22].

This paper presents a method to functionally reconstitute the voltage-gated potassium channel, KvAP, into GUVs. KvAP is a bacterial channel with high homology and strong functional and structural similarities to eukaryotic Kv channels [30]. Like the other members of the Kv family, the channel is tetrameric and consists of a central pore surrounded by four voltage sensors with each monomer contributing 2 helices (S5, S6) to the central pore, while the remaining 4 helices (S1–S4) form a voltage-sensing domain [31]. Using a partial dehydration/electroformation protocol [32] modified to allow a wider range of salt concentrations in the GUV interior [33], KvAP could be reconstituted into spherical Giant Unilamellar Vesicles (mean diameter∼10 µm) with no visible defects. GUVs could be formed from membranes with different compositions and the protein density was quantitatively characterized. Furthermore, patch clamp measurements of GUVs confirmed that after reconstitution the channel remained potassium-selective and voltage-gated. In the future, this method should be of great help in studying how the membrane influences the activity and distribution of voltage gated ion channels.

## Materials and Methods

### Purification and reconstitution in SUVs

Protein purification and reconstitution in Small Unilamellar Vesicles (SUVs) were performed following published procedures [30]. Note that a detailed protocol is presented in Text S1. Wild-type KvAP (containing a single endogenous cysteine, C247) cloned into the pQE60 vector (Qiagen, Hilden, Germany) was generously provided by Pr. R. MacKinnon (The Rockefeller University, New York, USA). KvAP was expressed in XL1-Blue cells with 0.4 mM IPTG (and 10 mM BaCl_2_ to block K^+^ permeation). Bacteria were solubilized in 40 mM DM (n-Decyl-Maltopyranoside (Anagrade), Affymetrix) and then purified using a polyhistidine-tag affinity column (HiTrap Ni-Sepharose, GE Healthcare). The hexa-histidine tag was cleaved by incubation overnight with thrombin (Roche; 2 units for 5 mg of protein), and 1 mM TCEP (Invitrogen) added to reduce cysteines prior to final purification on a Superdex 200 10/300 GL size exclusion column (GE Healthcare). An example of an elution profile is shown in Figure S1. Alexa Fluor 488 maleimide (Invitrogen) was used to fluorescently label the channel (Figure S2) and protein and fluorophore concentrations measured by absorbance spectroscopy (NanoDrop Spectrophotometer, Thermo Scientific). All experiments were performed using fluorescently marked protein (labeling ratio of 1.7±1.0 fluorophores per channel, or equivalently, 0.42±0.24 fluorophores per KvAP-monomer). The protein was reconstituted in 1,2-diphytanoyl-sn-glycero-3-phosphocholine (DPhPC) or Egg L-α-phosphatidylcholine (EPC): Egg L-α-phosphatidic acid (EPA) (9∶1 w∶w) (Avanti Polar Lipids, Alabama, USA) SUVs at a protein to lipid mass ratio of 1∶10. The final SUV concentration was approximately 10 mg/ml in a relatively low-salt buffer (5 mM KCl, 1 mM HEPES pH 7.4).

### GUV formation

GUVs containing KvAP were prepared using an electroformation protocol [32] modified to work with physiological buffers [33]. In this approach, a protein-lipid film is formed by partially dehydrating a solution of SUVs deposited onto electrodes. GUVs are then formed by rehydrating this film under an applied AC electric field (Figure S3). To control the lipid and protein composition of the initial SUV solution, protein-containing SUVs were mixed with SUVs containing the red fluorescent lipid, Texas Red® 1,2-dihexadecanoyl-sn-glycero-3-phosphoethanolamine (TR-DHPE; Invitrogen), 1,2-dioleoyl-sn-glycero-3-phosphoethanolamine-N-[methoxy(polyethylene glycol)-2000] (PEG-DOPE; Avanti Polar Lipids), or other lipids. 2–10 mM trehalose (generously provided by Pr. Lorenzo Cordone, Palermo, Italy) was added to the solution to protect the protein during the partial dehydration step [23]. The final lipid concentration of the SUV mixture was typically 3 mg/ml. Droplets of the SUV solution (∼0.35 µL/cm^2^) were deposited onto two parallel platinum wires (diameter = 0.5 or 0.8 mm; edge to edge distance = 2 mm) in a custom-made Teflon chamber (Figure S4), and the SUVs then allowed to dry for approximately 30 min. GUV formation buffer was then added to the chamber and an electric field applied during the rehydration of the film. For a “low salt” GUV formation buffer (5 mM KCl, 1 mM HEPES pH 7.3, 400 mM sucrose), the lipid film was rehydrated for approximately 2 hours under an applied AC voltage of 0.7 V RMS (Root Mean Square), 10 Hz. For a more physiological buffer (100 mM KCl, 10 mM HEPES pH 7.3, 200 mM sucrose), a voltage of 0.3 V RMS, 500 Hz was applied overnight. Further details of the GUV formation process are given in Text S2.

To prepare GUVs containing co-existing domains, DPhPC proteo-SUVs were mixed with SUVs containing DPPC (1,2-dipalmitoyl-sn-glycero-3-phosphocholine) and cholesterol to achieve a final molar ratio of DPhPC∶DPPC∶Cholesterol of 6∶2∶2 (protein∶lipid mass ratio of 1∶20), a composition previously shown to produce phase-separated GUVs at room temperature [34]. GUVs were prepared using a low salt buffer and to avoid phase separation during GUV formation, the deposition of SUVs, drying and electroformation were all performed at 50°C. This elevated temperature is not expected to lead to significant oxidation of these lipids [35], while KvAP is derived from a hyperthermophilic archaeon which grows optimally at 95°C [30].

### GUV imaging

The growth of GUVs on the wires was observed using phase contrast microscopy on a Zeiss Axiovert 135 microscope (LWD Zeiss Achroplan 40× air objective, 0.65 NA). For more detailed observations of vesicle morphology and fluorescence quantification, GUVs were transferred to a chamber containing a solution of glucose, NaCl or KCl and HEPES (pH 7.4) with the same osmolarity as the GUV formation buffer. Confocal images were taken using a Nikon Eclipse TE 2000-E microscope with a D-Eclipse C1 confocal head with two laser lines (λ = 488 nm; λ = 543 nm) and a Nikon Plan Fluor 100× oil objective (1.3 NA).

### Quantitative image analysis

Confocal images of GUVs were used to measure the protein density in the GUV membranes and study vesicle unilamellarity. To calibrate the measured fluorescence intensity as a function of fluorophore density, a classic electroformation protocol [36] was used to prepare EPC GUVs containing known densities of either the green fluorescent lipid, 2-(4,4-difluoro-5,7-dimethyl-4-bora-3a,4a-diaza-s-indacene-3-pentanoyl)-1-hexadecanoyl-sn-glycero-3-phosphocholine (Bodipy-HPC; Molecular Probes), or the red fluorescent lipid, TR-DHPE. Confocal images of the GUV equatorial plane were analyzed with a Matlab (Mathworks, MA, USA) program to determine the membrane intensity profile, as detailed in the Results section and Text S4.

### Electrophysiology

The activity of reconstituted channels was studied using the patch-clamp technique [37]. Patch pipettes were pulled from borosilicate glass (KIMAX 51, I.D. = 0.7 mm, O.D. = 1 mm, Catalog #46481-1, Kimble Chase Corp., NJ) using a P-2000 puller (Sutter Instrument Company, Novato, CA). Tip diameters were typically between 1–4 µm with a resistance of between 2–5 MOhms. For measurements of channel gating, the patch pipette was filled with the bath solution (100 mM KCl, 4 mM HEPES (pH 7.2), ∼200 mM glucose). For ionic selectivity experiments the patch pipette was filled with a low [K^+^] solution (10 mM KCl, 90 mM NaCl, 4 mM HEPES (pH 7.2), ∼200 mM glucose); the correction for the liquid-junction potential was calculated using the Henderson equation [38]. Gigaseals were formed by gently aspirating (10–500 Pa) a piece of the GUV membrane into the patch pipette and voltage-clamp measurements were then made either in the GUV-attached or the “inside-out” geometry using an Axon Multiclamp 700B electrode Amplifier (Molecular Devices), and a PCI-6221 DAQ Card (National Instruments) controlled via LabView (National Instruments). Current traces were filtered at 1 kHz (4-pole Bessel) and sampled at 5 kHz, and membrane currents and voltages are presented with respect to the GUV interior (i.e. V = −V_pipette_; I = −I_pipette_). For ionic selectivity measurements, sections of current traces consistent with single channel gating events were identified, and a histogram of the current was fitted to two gaussian functions representing the “open” and “closed” current levels. The mean single-channel current and standard error of the mean were calculated for each membrane voltage. Voltage-gating was tested by holding the membrane voltage at −100 mV (30 seconds per cycle) before applying a transient voltage step (5.5 seconds duration) to a more positive voltage. The mean current during the interval from 0.5 s to 1 s after the beginning of the voltage-step was used to estimate the peak patch current and conductance. To characterize the relative concentration of the protein in the GUV and patch pipette, a ProSilica GC1380 camera (t_exp_ = 50 or 100 ms; Allied Vision Technologies, Germany) was used to record epi-fluorescence images of the protein (marked with Alexa Fluor 488) and the membrane (TR-DPPE, 0.125% by mol). All the electrophysiological data presented in the main text were obtained at room temperature (∼20°C) using fluorescently labeled KvAP reconstituted into DPhPC GUVs grown in 100 mM KCl.

## Results

The protocol to produce GUVs containing KvAP is presented before describing the characterization of protein incorporation and activity.

### GUVs preparation

GUVs containing reconstituted KvAP were prepared using a protocol based upon a SUV fusion/electro-formation method [32]. In this approach, a solution of SUVs containing the protein is deposited onto electrodes. The controlled partial dehydration of this solution causes the SUVs to fuse together to form a protein-lipid film consisting of membranes stacked parallel to the film surface. Buffer is then added, and in the presence of an applied alternating voltage, the membranes detach from the stack to form GUVs. Although the precise mechanism of electroformation is not yet fully understood [39], [40], Figure S3 summarizes the key steps in the fusion/electroformation approach. Experimentally, the challenge is to efficiently transform proteo-SUVs into GUVs while still maintaining the protein in a functional state. The GUV yield and state of the protein depends upon multiple parameters including the composition of the SUV solution, the level of dehydration, the salt concentration of the GUV growth buffer, and the frequency and amplitude of the applied electric field. During the trials used to refine the protocol, the growth of GUVs on the electrodes was followed with phase contrast microscopy (e.g. Figure S5) while the incorporation of protein into GUVs was monitored using fluorescently labeled KvAP. Conditions which provided consistent GUV yields are described in detail in the Materials and Methods, and the Text S2.

To confirm the method was suitable for different biophysical applications, GUVs were prepared with several different membrane compositions and different buffers encapsulated inside the GUVs.

Clearly, the GUV internal solution is especially important for studies of ion channels. For a relatively “low-salt” buffer (5 mM KCl, 1 mM HEPES pH 7.3, 400 mM sucrose), useful GUV yields could be obtained by applying a sinusoidal voltage of 0.7 V (RMS) and f = 10 Hz for approximately two hours. Preparing GUVs with “physiological” salt concentrations has traditionally been more difficult as salt can have a significant effect on the rehydration/swelling step of GUV formation [41]. However, it was recently reported that a higher frequency voltage (∼500 Hz) allowed the efficient electroformation of GUVs containing 100 mM salt [33]. For such a growth buffer (100 mM KCl, 10 mM HEPES pH 7.3, 200 mM sucrose), GUVs containing KvAP could be formed using a voltage of 0.3 V (RMS), f = 500 Hz overnight.

**Figure 2:**
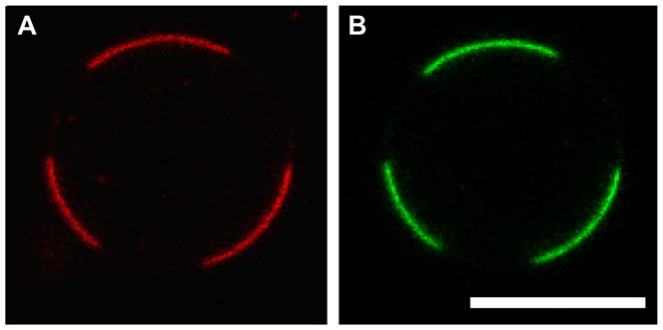
Partitioning of KvAP between co-existing liquid domains in GUVs. A) Confocal fluorescence image of TR-DHPE, a red fluorescent lipid that preferentially segregates into the liquid disordered phase. B) KvAP (green) is enriched in the same membrane domains. The GUV was formed from a lipid mixture of DPhPC, DPPC, and Chol (6∶2∶2 by mole with 0.25% of TR-DHPE) and KvAP was included at a protein to lipid ratio of 1∶20 (by mass). The gain has been adjusted to show the membrane more clearly. Scale bar: 5 µm.

Several lipid compositions were also tested. Both DPhPC and EPC∶EPA (9∶1 by mole) proteo-SUVs allowed the production of a high yield of GUVs. Confocal images of labeled KvAP in these vesicles (Figure 1B), showed that the protein was homogeneously distributed in the membrane. However, the reconstitution of Kv channels into GUVs with co-existing domains is also of interest. For example, the partitioning of Kv channels between membrane domains probes protein-membrane interactions and could be used to test whether lipid interactions can contribute to the localization of Kv channels in cells. As a proof of principle, GUVs were prepared containing a mixture of DPhPC∶DPPC∶Chol (6∶2∶2 by mole), with KvAP and 0.5% of TR-DHPE. DPhPC∶DPPC∶Chol has a well characterized phase diagram [34], and membranes with this composition (6∶2∶2) exhibit liquid ordered and liquid disordered domains at room temperature. TR-DHPE has been shown to preferentially segregate into liquid disordered phases [42]. Figure 2 shows a confocal image of a GUV containing co-existing domains. The protein (green) colocalizes with the TR-DHPE, which is consistent with enrichment of the channel in the liquid disordered phase.

Although the yield of GUVs depended on the exact parameters used, GUV formation was generally quite efficient with hundreds of GUVs per electro-formation chamber. The average GUV diameter also depended strongly on the exact parameters used, but was typically of the order of 10 µm (see Figure S6 for an example of a size distribution histogram for GUVs grown using EPC-EPA SUVs and the “low-salt” buffer). The unilamellarity of the vesicles was characterized by fluorescence microscopy of GUVs labeled with 0.5% (by mole) TR-DHPE. 1% by mole of PEG-DOPE was also added, as this has been reported to help produce defect-free GUVs [43]. Approximately half of the objects transferred from the electroformation chamber to an observation chamber were spherical with a single membrane visible (Figure S7). To determine if these vesicles were really unilamellar or had several membranes stuck closely together, their fluorescence intensity was compared to the fluorescence intensity of lipid-only GUVs prepared using a classical method known to produce unilamellar vesicles (see Material and Methods). As detailed in Text S3, the fluorescence of nearly all of these vesicles was indeed consistent with a single membrane (see Figure S8).

The following sections described in detail studies of the incorporation and activity of the protein.

### Protein density in GUVs

The channel density in GUVs was determined by measuring the fluorescence intensity of the membrane in confocal images of the vesicle equatorial plane. The fluorescence signal depends upon the protein density, intensity per fluorophore and the effective area of the GUV membrane within the confocal volume. Following the approach of Galush et. al. [44], the fluorescence signal was calibrated using the green fluorescent lipid, Bodipy-HPC, as a reference fluorophore that could be incorporated into membranes at known density. GUV fluorescence intensity was related to fluorophore density in the membrane through measurements of GUVs containing the reference fluorophore. Homogenous solutions of KvAP-Alexa and the reference fluorophore were then used to account for the relative intensity per fluorophore.

**Figure 3:**
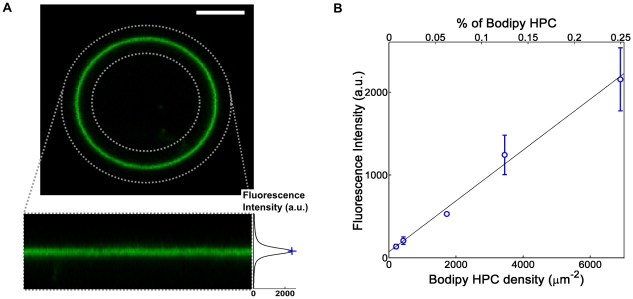
Calibration of GUV confocal fluorescence. A) Confocal images of the equatorial plane of GUVs containing Bodipy-HPC (top) were transformed into a coordinate system centered on the membrane contour (bottom) in order to calculate the membrane intensity profile (right). Membrane fluorescence intensity was characterized by the profile maximum (blue cross). Scale bar: 5 µm. B) Membrane fluorescence intensity as a function of Bodipy-HPC density. Each point represents ∼10 vesicles (except for the third point (density 1800 µm^−2^) which represents a single vesicle) and error bars show the sample standard deviation. The slope of this calibration curve, *M_ref_*, relates membrane fluorescence intensity to fluorophore density.

To determine membrane intensity, confocal images of GUVs containing known densities of Bodipy-HPC were analyzed using Matlab, as shown in Figure 3A. Note that for all quantitative measurements of fluorescence, the effect of confocal parameters and laser intensity were carefully taken into account (see details in Text S4). As expected, the measured membrane fluorescence intensity of reference GUVs was proportional to fluorescent lipid density (Figure 3B). Thus, the slope of this calibration curve, *M_ref_*, could be used to convert membrane fluorescence intensity to fluorophore density.

These measurements of the reference fluorophore were then related to the protein through the relative intensity of the two fluorophores. *F*, the fluorescence intensity of a molecule of Alexa-488 linked to KvAP relative to a molecule of Bodipy-HPC, was determined by measuring the fluorescence intensity of bulk solutions (SUVs and detergent micelles) with the confocal microscope. Following this calibration, the protein density in GUVs, *D*, could be deduced from the intensity of proteo-GUVs membrane, *I*, using the equation,
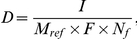
where *N_f_* is the number of Alexa 488 fluorophores per protein. This calibration method is described in detail in Text S4.

**Figure 4:**
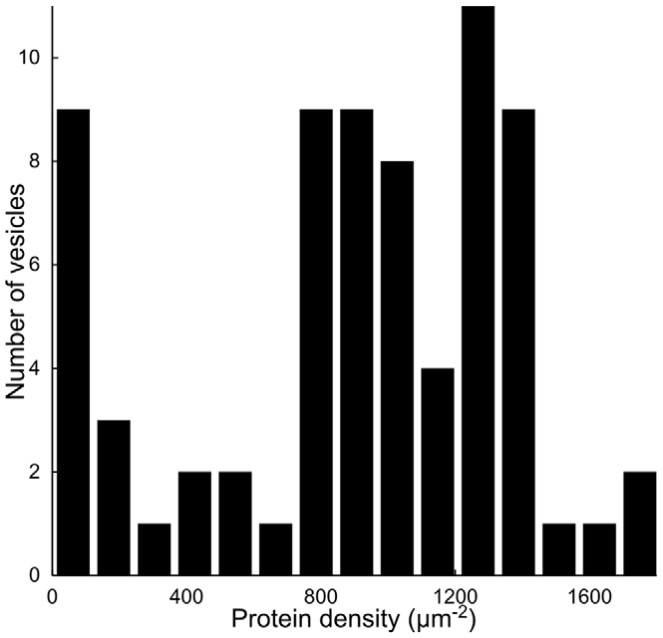
Histogram of protein density in GUVs. GUVs were formed using a low salt buffer (5 mM KCl, 1 mM HEPES, 400 mM sucrose) and protein density measured via quantitative confocal fluorescence (described in Text S4). The average protein density of the GUVs (1000±700 proteins/µm^2^) is comparable to the protein density of the (EPC∶EPA) SUVs from which they were formed (1600±400 proteins/µm^2^).

This technique was used to measure the GUV protein density. Figure 4 shows a histogram of protein density of 72 GUVs grown in low salt buffer and prepared from SUVs containing 1600±400 proteins/µm^2^ (measured via absorption spectroscopy as described in Text S4). The distribution is fairly broad but the average GUV protein density of 1000±700 proteins/µm^2^ is comparable to the protein density of the SUVs from which they were prepared. While a few GUVs did not contain detectable level of channels, KvAP was present in the vast majority with some containing well in excess of 1000 proteins/µm^2^. Furthermore, the average density of channels in GUVs could be controlled by adjusting the protein-to-lipid ratio of the SUVs.

Compared to GUVs prepared with a “low-salt” buffer, GUVs grown in the “physiological” buffer showed a larger variation in the density of incorporated protein. In particular, a small fraction of GUVs contained very high protein densities, while a considerable fraction of GUVs contained practically no protein (see Figures S9 and S10).

### Channel Activity

The fluorescent measurements described in the previous section showed that channels could be incorporated into GUVs. To determine if these channels were functional, their activity was studied using the well-established patch-clamp technique [37]. Briefly, a patch of GUV membrane was aspirated into a clean patch-pipette with an internal tip diameter of 1 to 4 µm. Adhesion between the membrane patch and pipette walls frequently lead to the formation of a high resistance “gigaohm seal”, thereby permitting measurements of membrane current through the patch at a fixed voltage (voltage-clamp).

**Figure 5:**
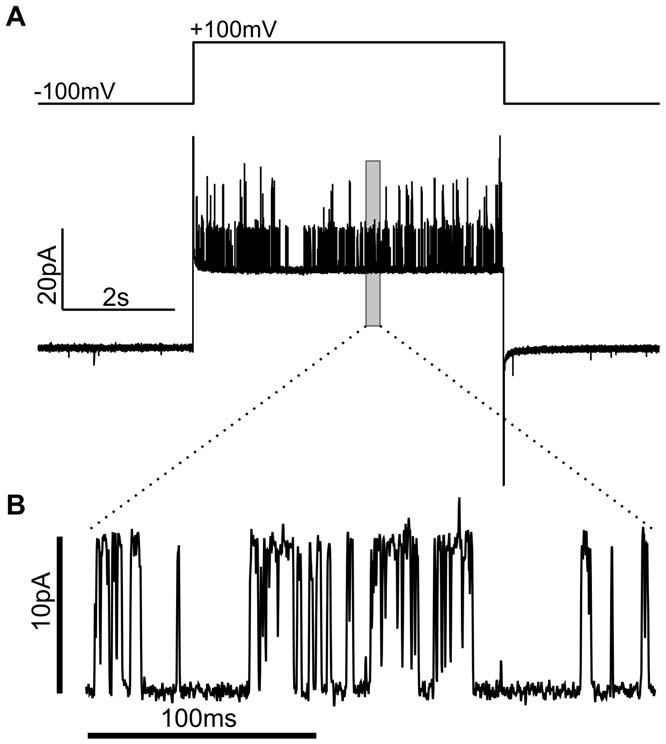
Activity of GUV Membrane Patches. A) GUV membrane patch current in response to an applied voltage step. Patch and bath solutions were both 100 mM KCl, 4 mM HEPES, pH 7.2, and the patch was formed from a DPhPC GUV containing fluorescent KvAP. B) Section of the trace showing distinct jumps in conductance (∼100 pS) that are consistent with the opening and closing of individual channels.

Figure 5 shows an example of such a current trace recorded from a membrane patch from a DPhPC proteo-GUV grown in high-salt buffer (see Figure S11 for an example of a trace recorded from an EPC-EPA proteo-GUV grown in low-salt buffer). The recording was made under symmetric conditions with 100 mM KCl in patch pipette and bath. Upon jumping to +100 mV, rapid transitions between distinct current levels were observed, consistent with the opening and closing of individual channels with a conductance of ∼100 pS. In general, membrane patches showed far more channel events at +100 mV than at −100 mV. This suggests that the channels in the patch membrane are both voltage-gated (as described in detail below) and preferentially oriented. Because the open probability of KvAP increases with voltage [30], the opening of channels at more positive voltages implies that they are inserted “physiologically” (i.e. with their intra-cellular domain facing into the GUV interior).

**Figure 6:**
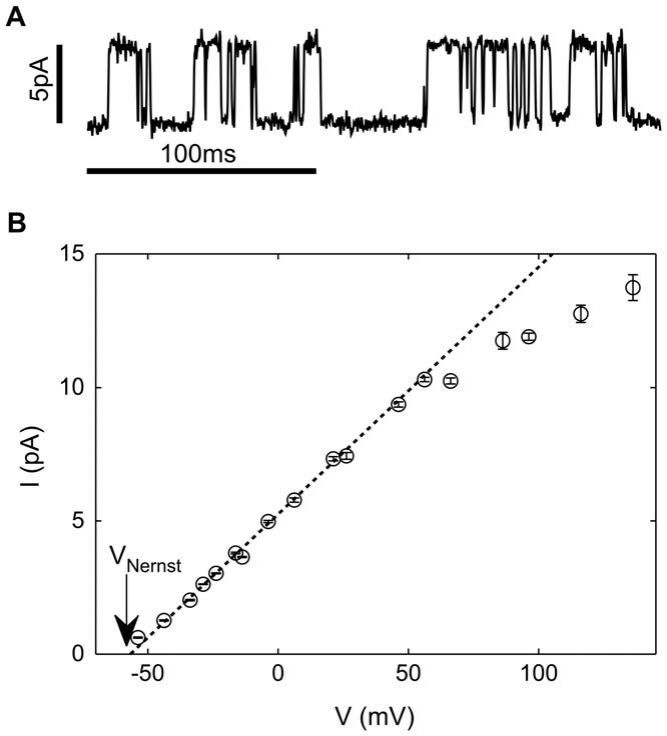
Ionic selectivity. A) Single channel current driven by ionic diffusion ([K+]_bath_ = 100 mM, [K+]_pipette_ = 10 mM, V_Nernst_ = −58 mV) overcome a small negative applied membrane potential (V = −4 mV). B) Dependence of single channel current, I, on membrane voltage, V. A linear fit for negative voltages (dotted line) gives a conductance of 93±2 pS and reversal voltage of −57±3 mV.

To characterize ionic selectivity, experiments were conducted in which 90% of the KCl in the pipette solution was substituted with NaCl. A potassium channel would block the diffusion of Na^+^ ions out of the pipette ([Na^+^]_pipette_ = 90 mM≫[Na^+^]_bath_), while allowing the diffusion of K^+^ into the pipette ([K+]_bath/GUV_ = 100 mM, [K+]_pipette_ = 10 mM, V_Nernst_ = −58 mV). Thus, even when the voltage across the membrane is zero, current should flow through a potassium channel as K+ ions diffuse into the pipette. As shown in Figure 6A, the K^+^ gradient drove current through the channels even when opposed by a small negative voltage (−4 mV). The dependence of mean single-channel current on applied voltage is shown in Figure 6B. The combination of a positive voltage and the concentration gradient drives large currents, while at negative voltages the current reduces as the potential opposes diffusion. A sufficiently negative voltage should reverse the current, but this was challenging to observe as channel events became increasingly rare at more negative voltages (consistent with voltage-dependent gating). However, the channels must be strongly selective as outward channel currents were observed at fairly negative voltages (e.g. V∼−50 mV). A linear fit for the range, V<0, yielded a conductance of 93±2 pS and reversal voltage of −57±3 mV (Figure 6C), which is within experimental error of the Nernst potential for potassium. These observations imply that KvAP in GUVs is highly-selective for potassium, consistently with its earlier characterization in BLMs [30].

**Figure 7:**
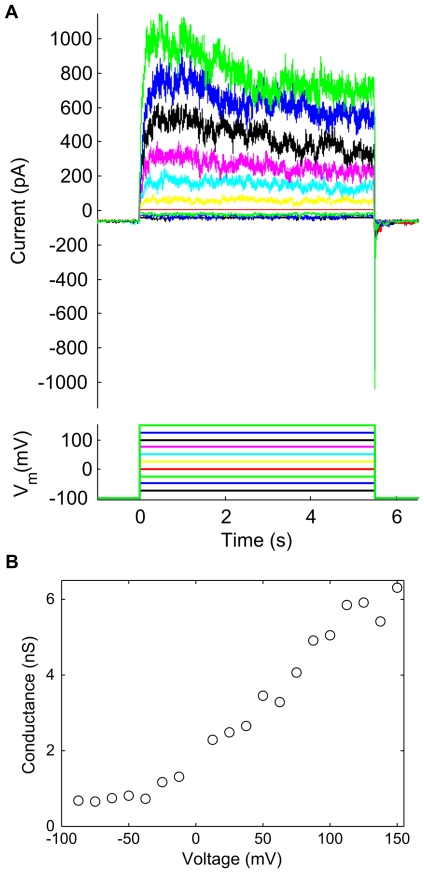
Voltage-dependent gating. A) Response of patch membrane current to a transient step in voltage. Pipette and bath solutions both contained 100 mM KCl, and the membrane was held for 30 seconds at −100 mV between successive voltage steps. Membrane current was filtered at 500 Hz. B) Peak conductivity as a function of voltage.

The voltage-dependent gating of channels was studied by measuring the current induced by a transient step in voltage. To characterize the ensemble behavior, these experiments were performed using larger patch pipettes (diameter of ∼3–4 µm) so as to obtain membrane patches containing more channels. Patch and bath solutions both contained 100 mM KCl, and the patch was held at −100 mV between voltage steps. As shown in Figure 7A, stepping to a positive voltage caused channels to open rapidly. The moderate decrease in current during the step is consistent with some channels entering the inactivate state [45]. Finally, channels rapidly closed at the end of the step as the voltage returned to −100 mV. The tendency for more channels to open with increasing voltage is clearly evident in the dependence of peak membrane conductance on applied voltage (Figure 7B). For V<−25 mV, very few channels were open and the conductance was dominated by the leak current of patch. In contrast, at very positive voltages (V≥100 mV), approximately 50 channels were open on average (assuming a channel conductance of ∼100 pS). Furthermore, the large fluctuations in membrane current imply that the open probability for individual channels remains well below 1. The observed voltage-dependent gating is not identical to the reported KvAP gating in DPhPC BLMs [45]. In particular, the increase in channel open probability occurs both more gradually and at more positive voltages, while channel inactivation is both markedly slower and incomplete. However, despite differences from BLM measurements, the activity of KvAP in GUVs is indeed voltage-dependent.

**Figure 8:**
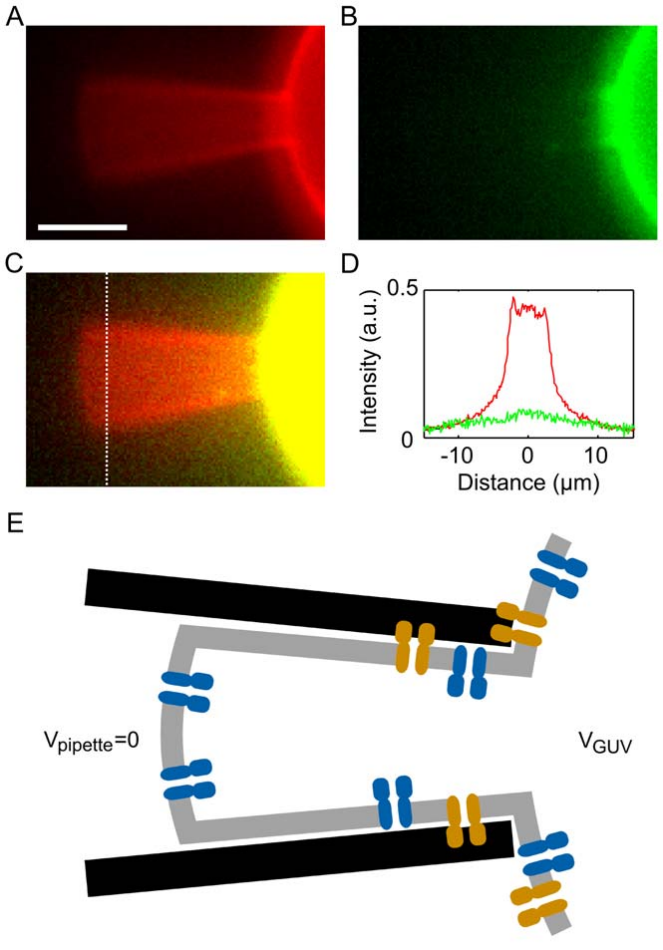
Protein concentration in the patch membrane. A–B) Epi-fluorescence image of the patch pipette and GUV showing the lipid (A, red) and KvAP (B, green). Red and green channels are scaled to be equal on the GUV body. C) Merged image with contrast increased to show the absence of protein (green) fluorescence in the patch. Scale Bar: 5 µm. D) Fluorescence intensity across the patch membrane (dotted line in C) of lipid (red) and KvAP (green). E) Schematic illustrating how adhesion between the membrane and pipette could favor the “correct” (blue; intracellular domain facing into the GUV interior) insertion over the “reverse” insertion (orange). The larger intracellular domain of the “reverse” insertion may stick more easily to the patch pipette walls, thereby excluding it from the patch membrane.

While it is tempting to compare the number of active channels in a membrane patch to the density of channels in the GUVs, studies have shown that the composition of the cell and patch membrane can differ [46]. To compare the composition of patch and GUV membranes, patch-clamped GUVs were imaged via epi-fluorescence as shown in Figure 8. Fluorescence from the channels (green) and membrane (TR-DHPE ; red) were scaled to be equal on the GUV body so as to permit direct comparison of the fluorescent protein/lipid concentration. The protein concentration in the patch membrane is clearly lower as protein fluorescence cannot be observed in the patch, while lipid fluorescence can be easily resolved. Epi-fluorescence is not ideal for making a quantitative estimate due to the strong fluorescent signal from the GUV, but the protein/lipid concentration must be at least 10 times lower in the patch membrane. This observation suggests that channels are strongly excluded from the patch membrane and prevents a simple comparison of patch and GUV membranes.

## Discussion

Despite the great interest in the reconstitution of transmembrane proteins into GUVs, progress has been challenging because many protocols that have a high GUV yield use conditions that could damage proteins. To date, two main strategies have been employed. In the first approach, lipid-only GUVs are first prepared and the protein is then incorporated afterwards. Efforts have been made to introduce the protein by mixing GUVs with protein-detergent micelles [24], and by fusing proteo-SUVs to GUVs either spontaneously [25], or with the aid of charged lipids and fusion peptides [47]. While this strategy protects the protein from extreme conditions, the final density of proteins in GUVs has typically been quite low and difficult to control, while conditions must be finely tuned to avoid destroying the GUVs when they are exposed to the detergent or fusion-enhancing charged lipids or peptides [22].

In the second approach, GUVs are formed directly from a membrane film that already contains the protein [32]. The membrane stack has typically been formed by partial dehydration of a solution of proteo-SUVS, while applying an AC electric field during rehydration (electroformation) can greatly boost the yield of unilamellar defect-free vesicles [48]. An obvious drawback of this approach is the difficulty of forming a membrane film without damaging the protein, although sugars (e.g. sucrose or trehalose) can be added to stabilize the protein during the dehydration step [23]. This SUV dehydration/electroformation approach has been successfully used to reconstitute a number of membrane proteins into GUVs for studies including the affinity of proteins for lipid rafts [49], the effect of pump activity on membrane fluctuations [50], the effect of membrane composition and thickness on protein mobility [51], and cell-cell adhesion [43].

The results presented in this paper indicate that the SUV dehydration/electro-formation method is an effective method to reconstitute KvAP into GUVs. Starting with a modest amount of material (∼10 µg of SUVs), the method yielded hundreds of GUVs per chamber with a typical GUV diameter of ∼10 µm. Like many GUV preparation methods, the protocol produced a fraction of multi-lamellar objects. However, most GUV experiments manipulate or at least analyze GUVs individually so multi-lamellar objects can be readily detected (and excluded) by fluorescence microscopy. Importantly, the protocol does not require any specific membrane components (e.g. charged lipids, peptides, etc.), and GUVs were grown with several different membrane compositions simply by changing the composition of the initial SUV solution. This ability to vary membrane composition is very important for studies of protein-membrane interactions. Finally, even though electro-formation has traditionally been applied using low-salt buffers, GUVs could be effectively prepared in buffers containing physiological salt concentrations by applying a voltage with a higher frequency (∼500 Hz) [33]. Thus, this approach allows the formation of GUVs without imposing serious constraints either on GUV composition or internal solution.

To study the incorporation of protein in GUVs, a calibration method was developed to allow quantitative measurements of the protein density in GUVs using only a confocal fluorescence microscope. Images of the labeled channel in GUVs showed that the protein was homogeneously distributed at optical length-scales, while measurements of protein diffusion (reported in [52]) were also inconsistent with the formation of large aggregates. However, the protein incorporation depended on whether GUVs were grown using “low-salt” or “physiological” buffer. For GUVs prepared using a low salt buffer, the average protein density in GUVs was comparable to the density of the SUVs from which they were formed. Furthermore, some batches of GUVs had a very small variation in the concentration of protein in individual GUVs. Thus, under these conditions the protein density in GUVs could readily be controlled by varying the density in SUVs. In contrast, GUVs formed in “physiological” buffer exhibited a very broad distribution of protein densities with some GUVs containing practically no protein while others contained densities higher than the initial concentration in SUVs. Such compositional heterogeneity has previously been observed in GUVs formed from immiscible lipid mixtures when either the film deposition or electroformation is performed at too low a temperature [53]. As both low and “physiological” salt GUVs are prepared from the same protein-lipid film, it seems likely that the heterogeneity occurs during the growth of GUVs from the film rather than during the deposition. Practically, the compositional heterogeneity of GUVs formed with physiological buffer can even be advantageous, as an experimenter can select GUVs with different protein densities from a single GUV batch. While more work is required to better understand and control protein incorporation, the present protocol produces GUVs with protein densities up to ∼1000 channels/µm^2^. The density of voltage-gated ion channels in cell membranes varies widely from close to zero (∼1 channel/µm^2^) to several thousand channels per square micron in specialized regions such as the nodes of Ranvier in the axon [1]. The channel density in the GUVs is comparable to this range and high enough to permit a wide range of biophysical measurements.

The activity of the reconstituted protein was tested via patch-clamp. These measurements showed that the channels in the GUV membrane were both selective for potassium and voltage-gated. Several factors could account for the interesting differences between KvAP dynamics in DPhPC GUVs and BLMs [45]. Amphipathic agents such as alcohols and anesthetics have been reported to modulate KvAP gating in BLMs [54], suggesting that the protein is sensitive to the membrane environment. Thus the difference in membrane tension between the GUV membrane patch and BLM [11], or the presence of residual alkane in the BLM, could modify channel gating. Additionally, the Alexa 488 label could have an effect, as a recent study of KvAP-DNA chimera in which ssDNA was attached to a site near the native cysteine (V240C) found that channel opening shifted to much more positive voltages with a less sharp voltage response [55]. While further work is required, the ability to use GUVs to study the ensemble behavior of channels while controlling membrane parameters should help greatly in understanding membrane/channel interactions.

The number of active channels in a membrane patch could not be directly compared to fluorescence measurements of the channel density in GUVs because KvAP was strongly excluded from the patch membrane. Indeed, in epi-fluorescence images of the membrane patch, the fluorescent lipid TR-DHPE was easily detected while there was no discernable signal from KvAP (Figure 8D). This observation illustrates just how different the composition and tension of the patch membrane can be from the source membrane [46], and this very different environment must be considered when comparing the behavior of channels in membrane patches and cells [56].

It was somewhat unexpected that the active channels in the patch membranes were inserted almost exclusively in the physiological orientation (e.g. cytoplasmic domain facing into the GUV interior). Although membrane proteins reconstituted into SUVs can have a net orientation, it seems reasonable that this would be scrambled during the SUV fusion and that GUVs would contain approximately equal amounts of both insertions [32]. Interestingly, previous patch-clamp studies of KcsA [57], [58] and KirBac [59] reconstituted into MLVs also reported that channels were predominantly inserted with this same orientation (cytoplasmic domain facing into the MLV interior). Figure 8E illustrates how this orientation could result from interactions between proteins and the patch pipette. During the formation of a “giga-seal”, the membrane tightly adheres to the patch pipette [46]. After adhesion to the glass, lipids remain quite mobile while proteins are effectively immobilized. In crystal structures of KvAP [31], KcsA [60], and KirBac [61], the channel does not project very far out from the extra-cellular side of the membrane, while the C-terminus of KcsA and KirBac form comparatively large intra-cellular domains (in available crystal structures of KvAP the C-terminus is disordered or truncated). Consequently, channels oriented with the intra-cellular domain facing out (“non physiological” orientation) may stick easily to the glass, while channels with the “physiological” orientation may be able to more easily enter/stay in the patch membrane. Although channels may indeed be preferentially oriented in MLVs and GUVs, this adhesion-based mechanism would account both for the reduced density and preferred orientation of channels observed in the patch membrane.

To conclude, we have presented a method to reconstitute functional Kv channels at a relatively high density in cell-sized, defect-free unilamellar vesicles. These GUVs should be of great help in unraveling important open questions concerning the biophysics of Kv channels. For example, the interaction between channels and lipids can be directly studied by observing the lateral distribution of ion channels in GUVs containing co-existing domains. Measurements of diffusion (for example using FCS or single particle tracking) should also give insight into the effects of membrane parameters on Kv channel mobility and clustering state [49]. Finally, the combination of patch-clamp measurements with GUV manipulation should be especially powerful for studying channel function while controlling the physical state of the membrane (tension, curvature, etc.). Thus, GUVs containing functional voltage-gated ion channels should prove a powerful tool for studying channel/membrane interactions so as to understand how the membrane influences ion channel activity, distribution and diffusion and modulates cellular excitability.

## Supporting Information

Figure S1 **Size Exclusion Column Profile of purified KvAP.** The peak at 11 ml corresponds to KvAP.
(TIF)

Figure S2 **SDS-PAGE of purified KvAP.** The left image shows the gel stained with Coomassie Blue while the image on the right image shows fluorescence from the same gel (prior to staining). (Lane 1: protein ladder; Lane 2: KvAP after labeling with Alexa 488; Lane 3: unlabelled KvAP). Samples were run on a 12% SDS-PAGE gel and approximately 50 mM of beta-mercaptoethanol was added to samples to reduce disulfide bonds. Under these conditions KvAP runs as a monomer with an apparent molecular weight of ∼28 kDa.
(TIF)

Figure S3 **Schematic of the electro-formation process.** Droplets containing SUVs are deposited on the electrode. Partial dehydration of the solution causes the SUVs to fuse to form a stack of membranes. Buffer is then added and an AC electric field applied. As the film swells, individual membranes detach from the stack to form GUVs.
(TIF)

Figure S4 **GUV electroformation chamber.** The two platinum electrodes are mounted in a teflon block with 3 wells. The top and bottom of the wells are sealed with microscope coverslips, and the two platinum wires are connected to a signal generator via alligator clips.
(TIF)

Figure S5 **Phase contrast image showing GUVs growing on the platinum electrode.** GUVs were grown from an EPC∶EPA proteo-SUV solution (lipid concentration of 2 mg/ml; 1∶10 protein to lipid mass ratio; 10 mM trehalose). The growth buffer was 100 mM KCl, 10 mM HEPES (pH 7.4), 2 mM EDTA, and 200 mM sucrose. GUVs were formed by growth overnight with an applied voltage of 0.8 V_RMS_, f = 500 Hz. The scale bar is 20 µm.
(TIF)

Figure S6 **Histogram of Proteo-GUV Diameters.** GUVs were formed from EPC∶EPA SUVs using a low salt buffer (5 mM KCl, 1 mM HEPES, 400 mM sucrose), and a histogram of GUV protein density for the same population is show in Figure 4. The GUVs have a mean diameter of 7.9 µm and standard deviation of 2.9 µm (N = 71).
(TIF)

Figure S7 **Representative image of objects harvested from the GUV growth chamber.** A) Confocal image of GUVs containing the red fluorescent lipid, TR-DHPE (0.5% by mole). B) Corresponding signal for KvAP (green; Alexa 488). The white arrows indicate examples of round vesicles with a single membrane. The fluorescence intensity of these “apparent GUVs” was then analyzed for unilamellarity. Note that the fluorescence intensity is brighter in the center of this image because of the extremely large field of view. Scale bar: 20 µm.
(TIF)

Figure S8 **Vesicle unilamellarity.** Histograms of membrane fluorescence intensity for GUV prepared with lipids only (Reference), proteins in low-salt buffer, and proteins in high-salt buffer. Each distribution was fitted to a gaussian function to determine the mean and standard deviation (pure lipid GUVs - Mean = 2331, Std Dev = 487, N = 96; low salt proteo-GUVs - Mean = 2291, Std Dev = 422, N = 67; 100 mM salt proteo-GUVs - Mean = 1884, Std Dev = 511, N = 75).
(TIF)

Figure S9 **Dependence of protein density on GUV preparation method.** Histograms of protein density for batches of GUVs prepared in low salt buffer (upper, N = 86) and high salt buffer (lower histogram, N = 88). Note these batches represent extreme cases showing the most homogenous and heterogeneous distributions. Low-salt GUVs were not always so uniform (e.g. Figure 4) while high-salt GUVs were not always so disperse (e.g. Figure S10).
(TIF)

Figure S10 **Variability of protein density in “high salt” GUVs is not due to multilamellarity.** Protein density histogram (N = 75) of the population of high salt proteo-GUVs presented in Figure S8 (which were shown to be unilamellar via lipid fluorescence). Although the GUVs are unilamellar, the protein density still varies between GUVs with very high protein densities in a couple of GUVs.
(TIF)

Figure S11 **Activity of Membrane Patch from a GUV formed with low-salt buffer.** A) GUV membrane patch current in response to an applied voltage step. Patch and bath solutions were both 100 mM KCl, 4 mM HEPES, pH 7.2, and the patch was formed from a EPC∶EPA (9∶1 by mole) GUV formed using low-salt buffer (5 mM) B) Section of the trace showing distinct jumps in conductance that are consistent with the opening and closing of individual channels.
(TIF)

Text S1 **KvAP Purification Protocol.** Procedures for the expression and solubilization, purification, fluorescent labeling and reconstitution into proteoliposomes of KvAP.
(PDF)

Text S2 **Proteo-GUV formation Protocol.** Methods used to produce GUVs with reconstituted KvAP, GUVs containing co-existing liquid domains and pure lipid GUVs.
(PDF)

Text S3 **Characterization of the Proteo-GUVs.** Description of methods used to characterized vesicle unilamellarity, size distribution and protein incorporation.
(PDF)

Text S4 **Measurement of Protein Density.** Explanation of the fluorescence calibration method, quantification of membrane fluorescence intensity, area of the GUV membrane in the confocal volume and fluorophores per protein.
(PDF)

## References

[1] Hille B (2001). Ion Channels of Excitable Membranes (3rd Edition).

[2] Marban E (2002). Cardiac channelopathies.. Nature.

[3] Ryan DP, Placek LJ (2010). Episodic neurological channelopathies.. Neuron.

[4] Kullmann DM (2010). Neurological channelopathies.. Annu Rev Neurosci.

[5] Lai HC, Jan LY (2006). The distribution and targeting of neuronal voltage-gated ion channels.. Nat Rev Neurosci.

[6] Bezanilla F (2008). Ion channels: from conductance to structure.. Neuron.

[7] Ramu Y, Xu Y, Lu Z (2006). Enzymatic activation of voltage-gated potassium channels.. Nature.

[8] Gamper N, Shapiro MS (2007). Regulation of ion transport proteins by membrane phosphoinositides.. Nat Rev Neurosci.

[9] Marsh D (2007). Lateral pressure profile, spontaneous curvature frustration, and the incorporation and conformation of proteins in membranes.. Biophys J.

[10] Reeves D, Ursell T, Sens P, Kondev J, Phillips R (2008). Membrane mechanics as a probe of ion-channel gating mechanisms.. Phys Rev E.

[11] Schmidt D, MacKinnon R (2008). Voltage-dependent K+ channel gating and voltage sensor toxin sensitivity depend on the mechanical state of the lipid membrane.. P Natl Acad Sci USA.

[12] Martens JR, O'Connell K, Tamkun M (2004). Targeting of ion channels to membrane microdomains: localization of Kv channels to lipid rafts.. Trends Pharmacol Sci.

[13] Lingwood D, Simons K (2010). Lipid rafts as a membrane-organizing principle.. Science.

[14] McMahon HT, Gallop JL (2005). Membrane curvature and mechanisms of dynamic cell membrane remodelling.. Nature.

[15] Phillips R, Ursell T, Wiggins P, Sens P (2009). Emerging roles for lipids in shaping membrane-protein function.. Nature.

[16] Miller C (1986). Ion channel reconstitution.

[17] Dilger JP, Benz R (1985). Optical and electrical properties of thin monoolein lipid bilayers.. J Membrane Biol.

[18] Requena J, Haydon D (1975). The Lippmann equation and the characterization of black lipid films.. J Colloid Interf Sci.

[19] Evans E, Rawicz W (1990). Entropy-driven tension and bending elasticity in condensed-fluid membranes.. Phys Rev Lett.

[20] Waugh RE, Song J, Svetina S, Zeks B (1992). Local and nonlocal curvature elasticity in bilayer membranes by tether formation from lecithin vesicles.. Biophys J.

[21] Heinrich V, Bozic B, Svetina S, Zeks B (1999). Vesicle deformation by an axial load: from elongated shapes to tethered vesicles.. Biophys J.

[22] Kahya N (2010). Protein-protein and protein-lipid interactions in domain-assembly: Lessons from giant unilamellar vesicles.. BBA-Biomembranes.

[23] Doeven MK, Folgering JHA, Krasnikov V, Geertsma ER, Den G (2005). Distribution, lateral mobility and function of membrane proteins incorporated into giant unilamellar vesicles.. Biophys J.

[24] Kreir M, Farre C, Beckler M, George M, Fertig N (2008). Rapid screening of membrane protein activity: electrophysiological analysis of OmpF reconstituted in proteoliposomes.. Lab Chip.

[25] Varnier A, Kermarrec F, Blesneac I, Moreau C, Liguori L (2010). A simple method for the reconstitution of membrane proteins into giant unilamellar vesicles.. J Membrane Biol.

[26] Mahendran KR, Kreir M, Weingart H, Fertig N, Winterhalter M (2010). Permeation of antibiotics through Escherichia coli OmpF and OmpC porins: screening for influx on a single-molecule level.. J Biomol Screen.

[27] Davidson M, Karlsson M, Sinclair J, Sott K, Orwar O (2003). Nanotube-Vesicle Networks with Functionalized Membranes and Interiors.. J Am Chem Soc.

[28] Yanagisawa M, Iwamoto M, Kato A, Yoshikawa K, Oiki S (2011). Oriented reconstitution of a membrane protein in a giant unilamellar vesicle: Experimental verification with the potassium channel KcsA.. J Am Chem Soc.

[29] Gassmann O, Kreir M, Ambrosi C, Pranskevich J, Oshima A (2009). The M34A mutant of Connexin26 reveals active conductance states in pore-suspending membranes.. J Struct Biol.

[30] Ruta V, Jiang Y, Lee A, Chen J, MacKinnon R (2003). Functional analysis of an archaebacterial voltage-dependent K+ channel.. Nature.

[31] Lee SY, Lee A, Chen J, MacKinnon R (2005). Structure of the KvAP voltage-dependent K+ channel and its dependence on the lipid membrane.. P Natl Acad Sci USA.

[32] Girard P, Pecreaux J, Lenoir G, Falson P, Rigaud JL (2004). A new method for the reconstitution of membrane proteins into giant unilamellar vesicles.. Biophys J.

[33] Meleard P, Bagatolli LA, Pott T (2009). Giant Unilamellar Vesicle Electroformation: From Lipid Mixtures to Native Membranes Under Physiological Conditions.. Method Enzymol.

[34] Veatch SL, Gawrisch K, Keller SL (2006). Closed-loop miscibility gap and quantitative tie-lines in ternary membranes containing diphytanoyl PC.. Biophys J.

[35] Morales-Penningston NF, Wu J, Farkas ER, Goh SL, Konyakhina TM (2010). GUV preparation and imaging: Minimizing artifacts.. BBA-Biomembranes.

[36] Mathivet L, Cribier S, Devaux P (1996). Shape change and physical properties of giant phospholipid vesicles prepared in the presence of an AC electric field.. Biophys J.

[37] Hamill OP, Marty A, Neher E, Sakmann B, Sigworth FJ (1981). Improved patch-clamp techniques for high-resolution current recording from cells and cell-free membrane patches.. Pflug Arch Eur J Phy.

[38] Barry PH, Lynch JW (1991). Liquid junction potentials and small cell effects in patch-clamp analysis.. J Membrane Biol.

[39] Angelova MI, Dimitrov DS (1986). Liposome electroformation.. Faraday Discuss Chem Soc.

[40] Shimanouchi T, Umakoshi H, Kuboi R (2009). Kinetic study on giant vesicle formation with electroformation method.. Langmuir.

[41] Akashi K, Miyata H, Itoh H, Kinosita K (1996). Preparation of giant liposomes in physiological conditions and their characterization under an optical microscope.. Biophys J.

[42] Baumgart T, Hunt G, Farkas ER, Webb WW, Feigenson GW (2007). Fluorescence probe partitioning between Lo/Ld phases in lipid membranes.. BBA-Biomembranes.

[43] Streicher P, Nassoy P, Barmann M, Dif A, Marchi-Artzner V (2009). Integrin reconstituted in GUVs: A biomimetic system to study initial steps of cell spreading.. BBA-Biomembranes.

[44] Galush WJ, Nye JA, Groves JT (2008). Quantitative fluorescence microscopy using supported lipid bilayer standards.. Biophys J.

[45] Schmidt D, Cross SR, MacKinnon R (2009). A gating model for the archeal voltage-dependent K(+) channel KvAP in DPhPC and POPE:POPG decane lipid bilayers.. J Mol Biol.

[46] Suchyna TM, Markin VS, Sachs F (2009). Biophysics and structure of the patch and the gigaseal.. Biophys J.

[47] Kahya N, Pecheur EI, de Boeij WP, Wiersma DA, Hoekstra D (2001). Reconstitution of membrane proteins into giant unilamellar vesicles via peptide-induced fusion.. Biophys J.

[48] Rodriguez N, Pincet F, Cribier S (2005). Giant vesicles formed by gentle hydration and electroformation: a comparison by fluorescence microscopy.. Colloid Surface B.

[49] Kahya N, Wiersma D, Poolman B, Hoekstra D (2002). Spatial organization of bacteriorhodopsin in model membranes. Light-induced mobility changes.. J Biol Chem.

[50] El Alaoui F, Lacoste D, Pecreaux J, Joanny JF, Prost J (2009). Membrane Tension Lowering Induced by Protein Activity.. Phys Rev Lett.

[51] Ramadurai S, Duurkens R, Krasnikov VV, Poolman B (2010). Lateral diffusion of membrane proteins: consequences of hydrophobic mismatch and lipid composition.. Biophys J.

[52] Domanov Y, Aimon S, Renner M, Quemeneur F, Toombes GES (2011). Mobility in geometrically confined membranes.. P Natl Acad Sci USA.

[53] Veatch SL, Keller SL (2005). Seeing spots: complex phase behavior in simple membranes.. BBA-Biomembranes.

[54] Finol-Urdaneta RK, McArthur JR, Juranka PF, French RJ, Morris CE (2010). Modulation of KvAP unitary conductance and gating by 1-alkanols and other surface active agents.. Biophys J.

[55] Wang A, Zocchi G (2011). Artificial Modulation of the Gating Behavior of a K Channel in a KvAP-DNA Chimera.. PLoS ONE.

[56] Zhang Y, Hamill OP (2000). On the discrepancy between whole-cell and membrane patch mechanosensitivity in Xenopus oocytes.. J Physiol.

[57] Molina M, Barrera F, Fernández A, Poveda J, Renart M (2006). Clustering and coupled gating modulate the activity in KcsA, a potassium channel model.. J Biol Chem.

[58] Chakrapani S, Cordero-Morales JF, Perozo E (2007). A Quantitative Description of KcsA Gating I: Macroscopic Currents.. J Gen Physiol.

[59] Cheng WWL, Enkvetchakul D, Nichols CG (2009). KirBac1.1: It's an Inward Rectifying Potassium Channel.. J Gen Physiol.

[60] Uysal S, Vásquez V, Tereshko V, Esaki K, Fellouse FA (2009). Crystal structure of full-length KcsA in its closed conformation.. Proc Natl Acad Sci U S A.

[61] Kuo A, Gulbis JM, Antcliff JF, Rahman T, Lowe ED (2003). Crystal Structure of the Potassium Channel KirBac1.1 in the Closed State.. Science.

