# Supplementary figures and images for: Functional Reconstitution of a Voltage-Gated Potassium Channel in Giant Unilamellar Vesicles
Source: PLoS One. 2011 Oct 6;6(10):e25529. doi: 10.1371/journal.pone.0025529 (PMC3188570; doi:10.1371/journal.pone.0025529)

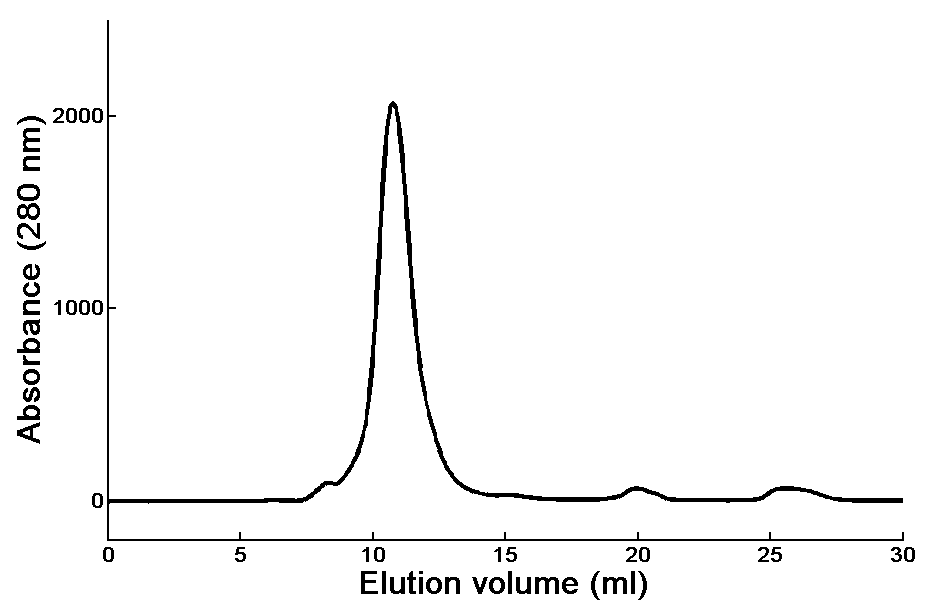

Supplement: Figure S1 — Size Exclusion Column Profile of purified KvAP. The peak at 11 ml corresponds to KvAP. (TIF) [file pone.0025529.s001.tif]

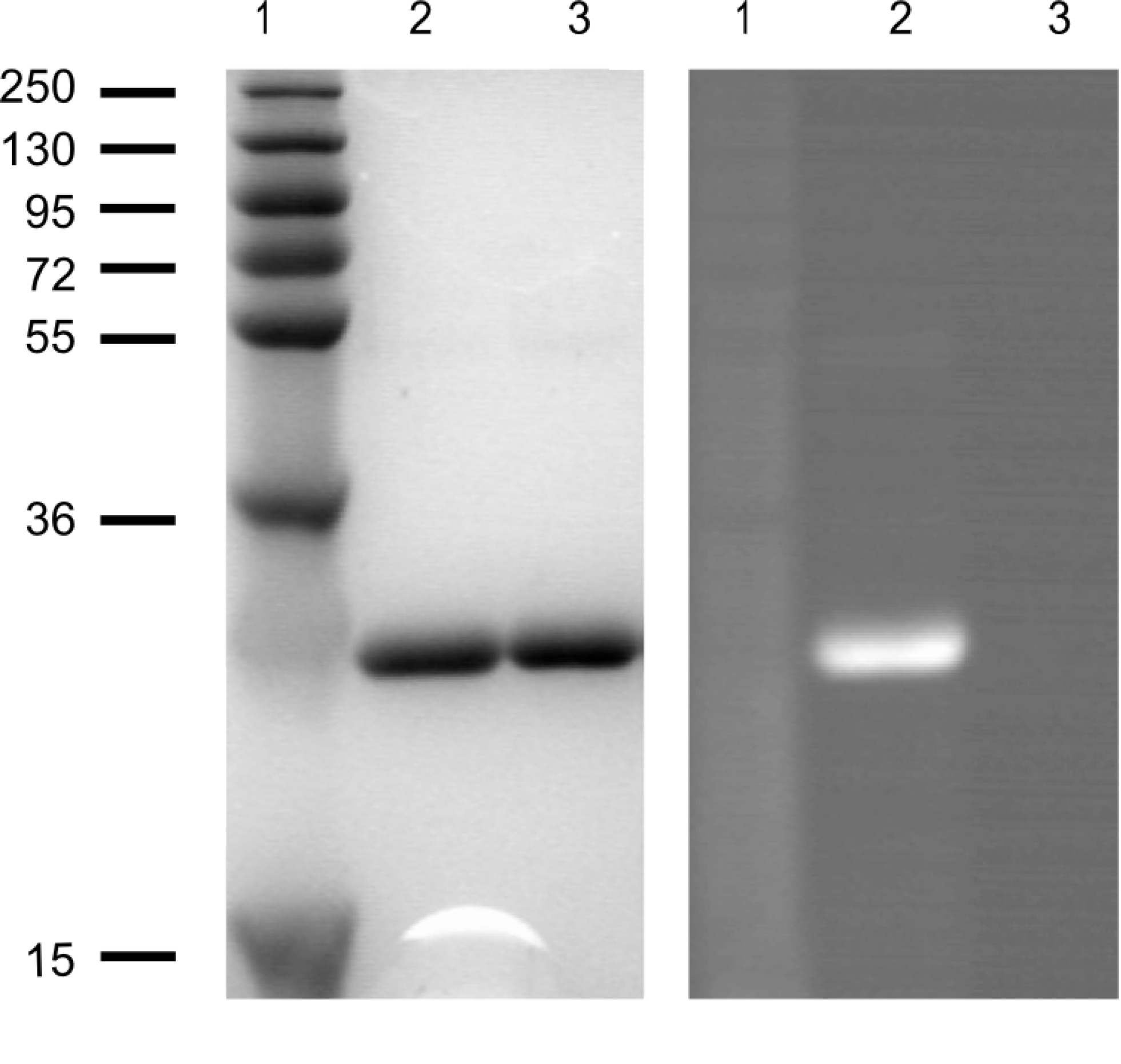

Supplement: Figure S2 — SDS-PAGE of purified KvAP. The left image shows the gel stained with Coomassie Blue while the image on the right image shows fluorescence from the same gel (prior to staining). (Lane 1: protein ladder; Lane 2: KvAP after labeling with Alexa 488; Lane 3: unlabelled KvAP). Samples were run on a 12% SDS-PAGE gel and approximately 50 mM of beta-mercaptoethanol was added to samples to reduce disulfide bonds. Under these conditions KvAP runs as a monomer with an apparent molecular weight of ∼28 kDa. (TIF) [file pone.0025529.s002.tif]

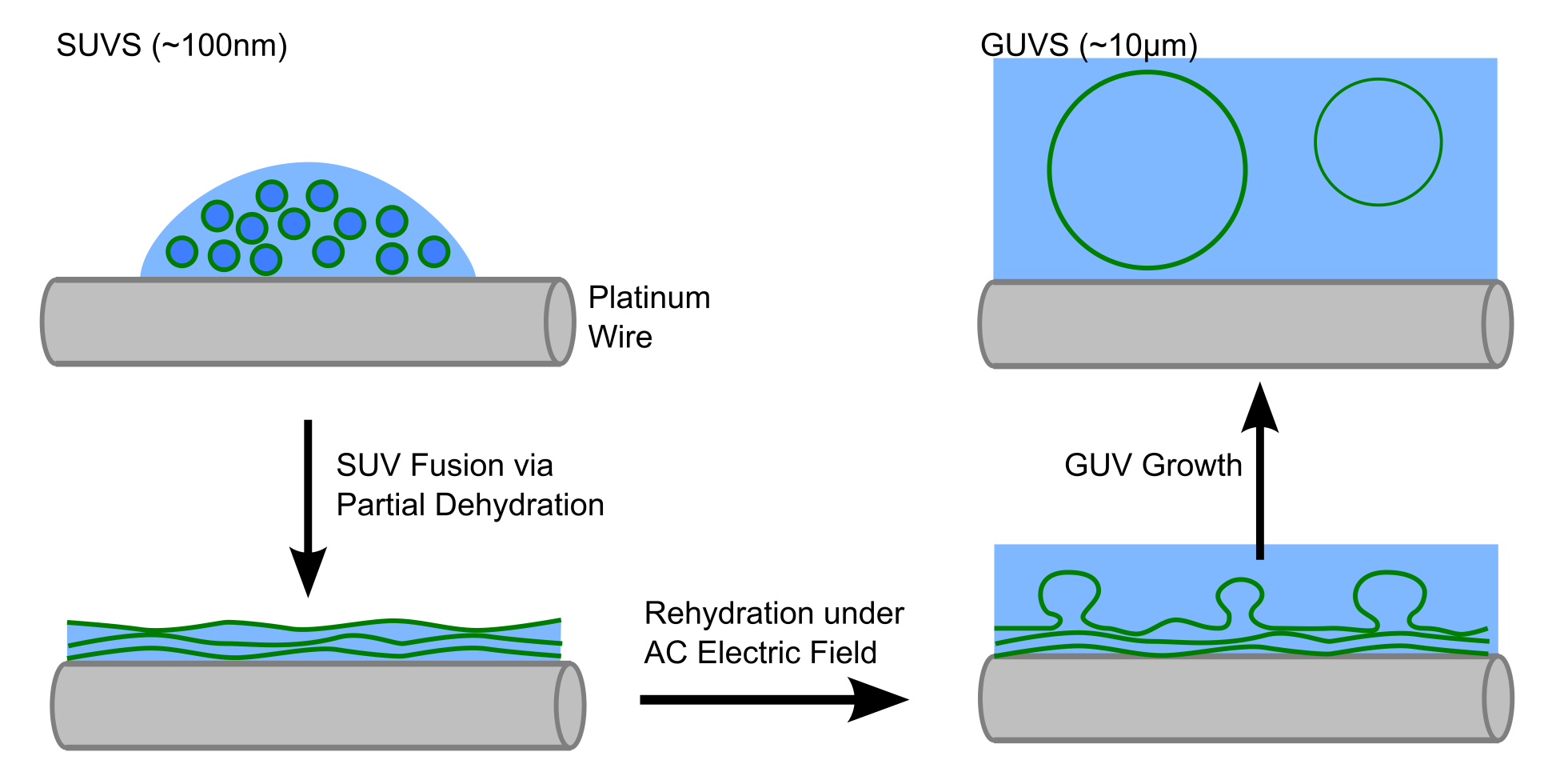

Supplement: Figure S3 — Schematic of the electro-formation process. Droplets containing SUVs are deposited on the electrode. Partial dehydration of the solution causes the SUVs to fuse to form a stack of membranes. Buffer is then added and an AC electric field applied. As the film swells, individual membranes detach from the stack to form GUVs. (TIF) [file pone.0025529.s003.tif]

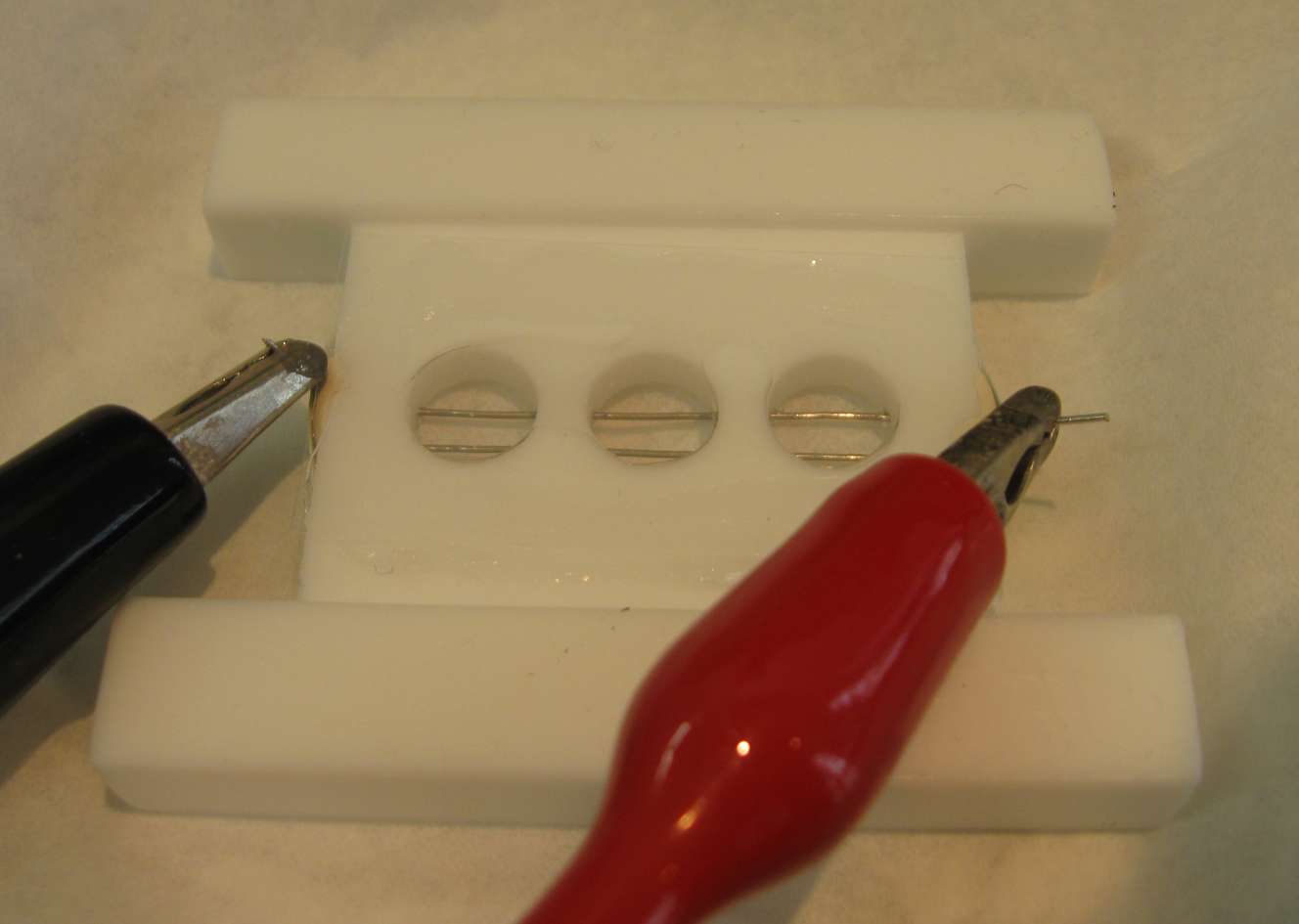

Supplement: Figure S4 — GUV electroformation chamber. The two platinum electrodes are mounted in a teflon block with 3 wells. The top and bottom of the wells are sealed with microscope coverslips, and the two platinum wires are connected to a signal generator via alligator clips. (TIF) [file pone.0025529.s004.tif]

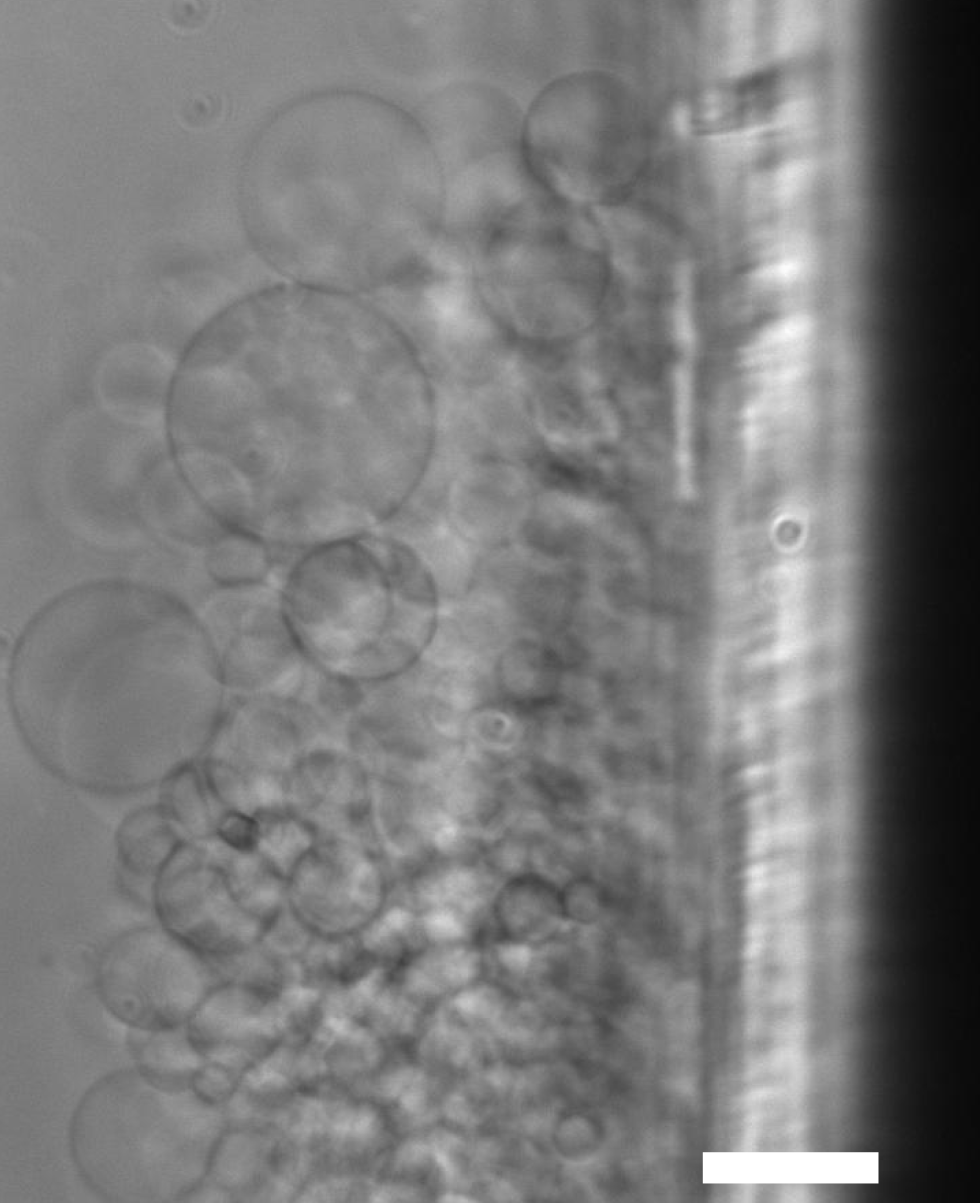

Supplement: Figure S5 — Phase contrast image showing GUVs growing on the platinum electrode. GUVs were grown from an EPC∶EPA proteo-SUV solution (lipid concentration of 2 mg/ml; 1∶10 protein to lipid mass ratio; 10 mM trehalose). The growth buffer was 100 mM KCl, 10 mM HEPES (pH 7.4), 2 mM EDTA, and 200 mM sucrose. GUVs were formed by growth overnight with an applied voltage of 0.8 VRMS, f = 500 Hz. The scale bar is 20 µm. (TIF) [file pone.0025529.s005.tif]

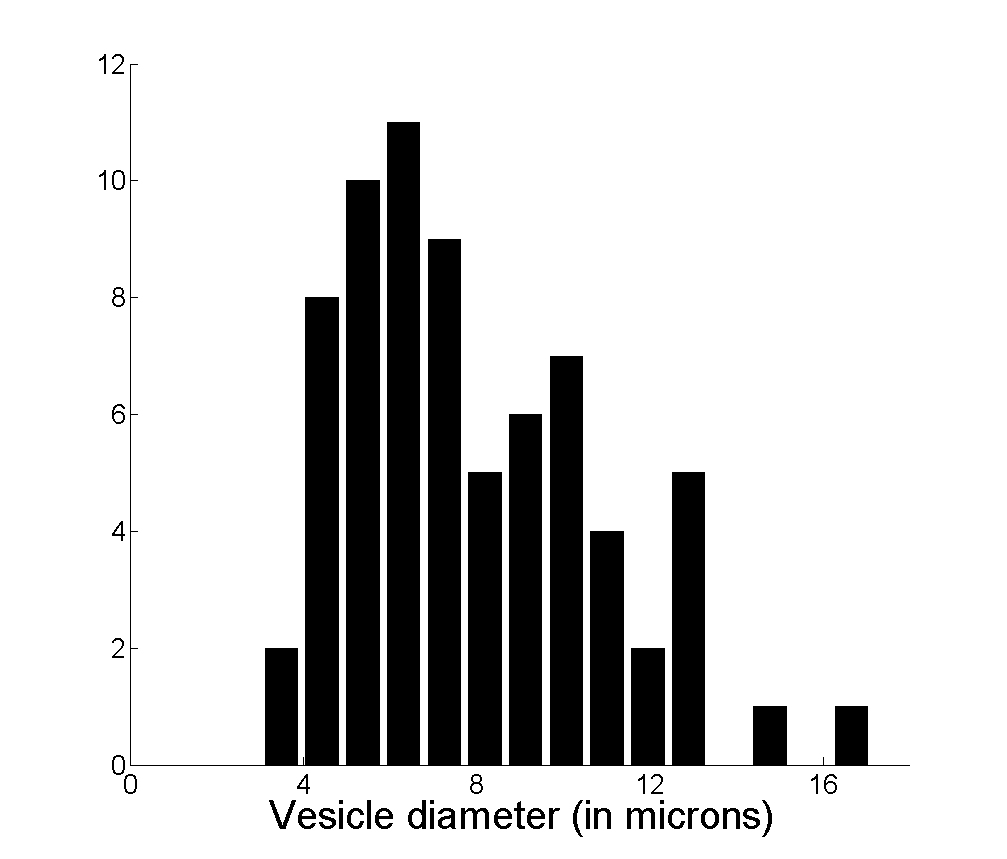

Supplement: Figure S6 — Histogram of Proteo-GUV Diameters. GUVs were formed from EPC∶EPA SUVs using a low salt buffer (5 mM KCl, 1 mM HEPES, 400 mM sucrose), and a histogram of GUV protein density for the same population is show in Figure 4. The GUVs have a mean diameter of 7.9 µm and standard deviation of 2.9 µm (N = 71). (TIF) [file pone.0025529.s006.tif]

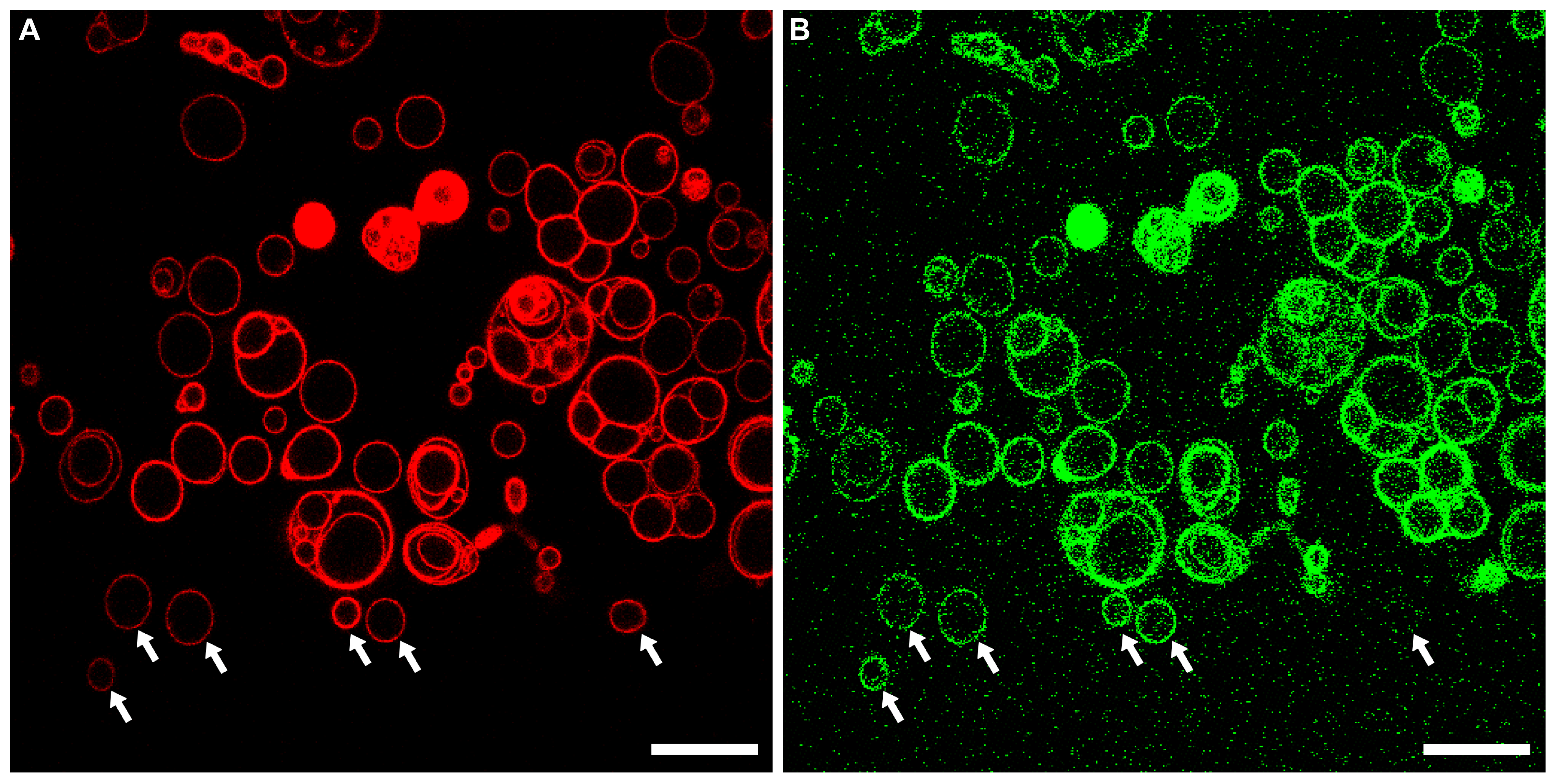

Supplement: Figure S7 — Representative image of objects harvested from the GUV growth chamber. A) Confocal image of GUVs containing the red fluorescent lipid, TR-DHPE (0.5% by mole). B) Corresponding signal for KvAP (green; Alexa 488). The white arrows indicate examples of round vesicles with a single membrane. The fluorescence intensity of these “apparent GUVs” was then analyzed for unilamellarity. Note that the fluorescence intensity is brighter in the center of this image because of the extremely large field of view. Scale bar: 20 µm. (TIF) [file pone.0025529.s007.tif]

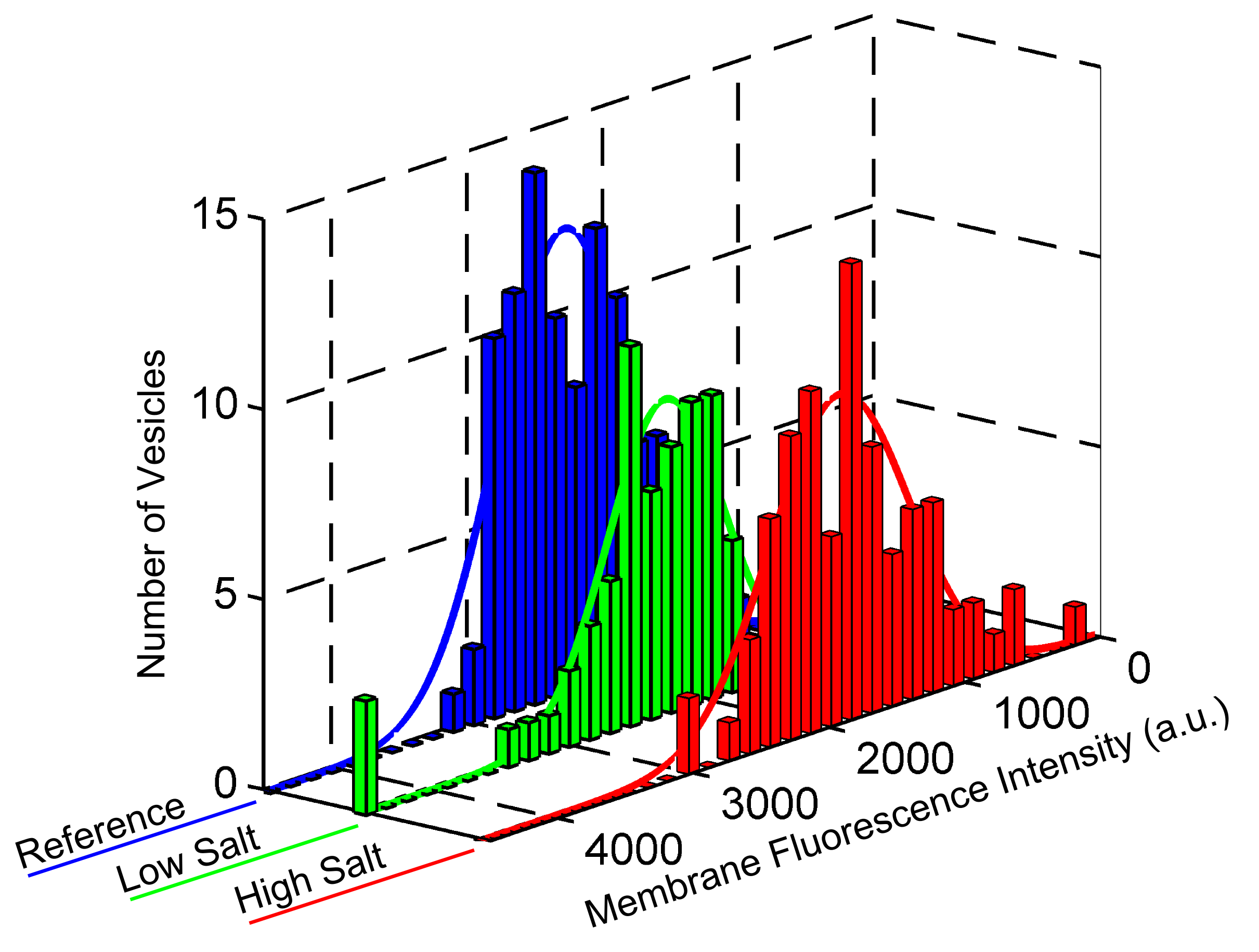

Supplement: Figure S8 — Vesicle unilamellarity. Histograms of membrane fluorescence intensity for GUV prepared with lipids only (Reference), proteins in low-salt buffer, and proteins in high-salt buffer. Each distribution was fitted to a gaussian function to determine the mean and standard deviation (pure lipid GUVs - Mean = 2331, Std Dev = 487, N = 96; low salt proteo-GUVs - Mean = 2291, Std Dev = 422, N = 67; 100 mM salt proteo-GUVs - Mean = 1884, Std Dev = 511, N = 75). (TIF) [file pone.0025529.s008.tif]

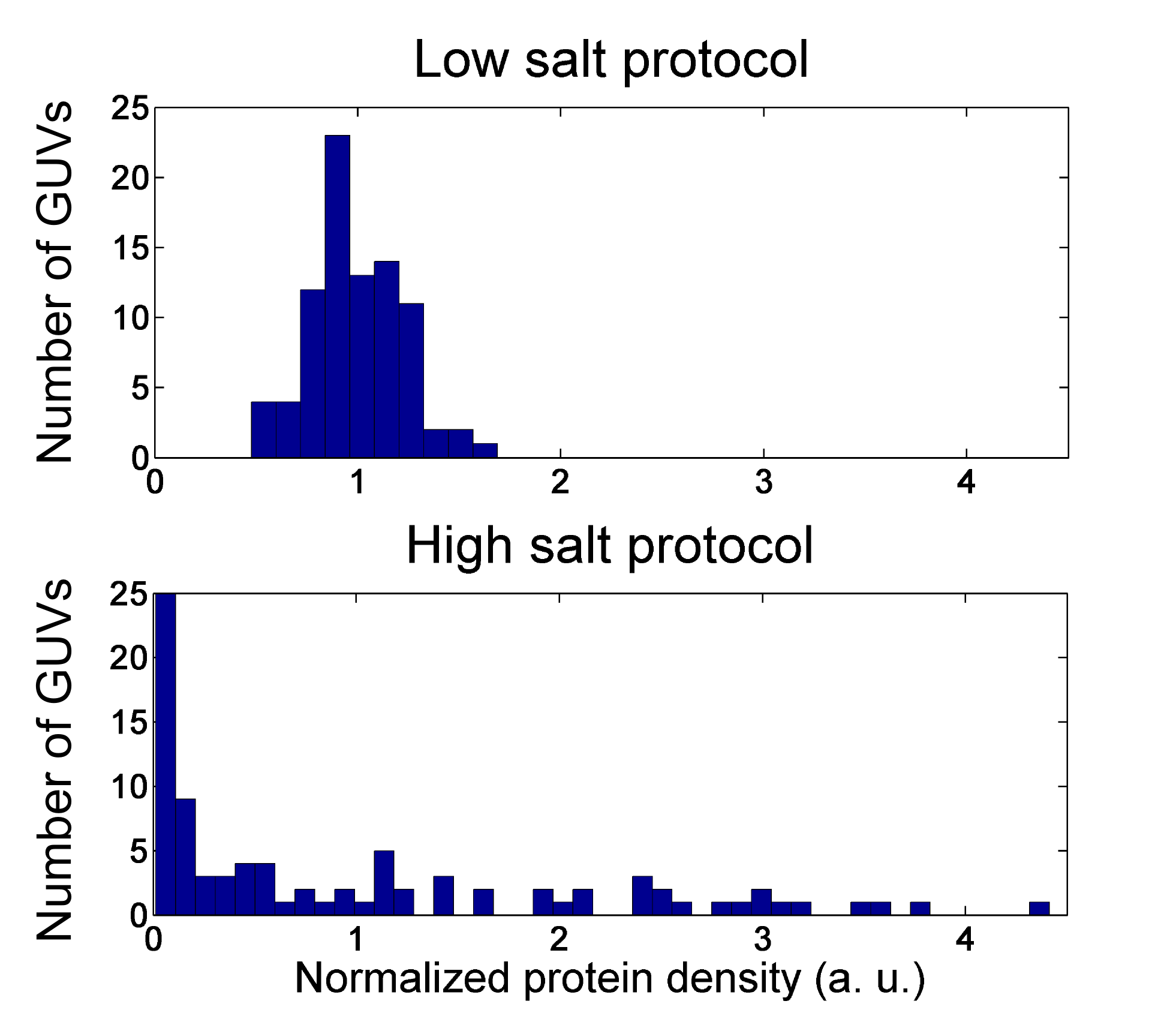

Supplement: Figure S9 — Dependence of protein density on GUV preparation method. Histograms of protein density for batches of GUVs prepared in low salt buffer (upper, N = 86) and high salt buffer (lower histogram, N = 88). Note these batches represent extreme cases showing the most homogenous and heterogeneous distributions. Low-salt GUVs were not always so uniform (e.g. Figure 4) while high-salt GUVs were not always so disperse (e.g. Figure S10). (TIF) [file pone.0025529.s009.tif]

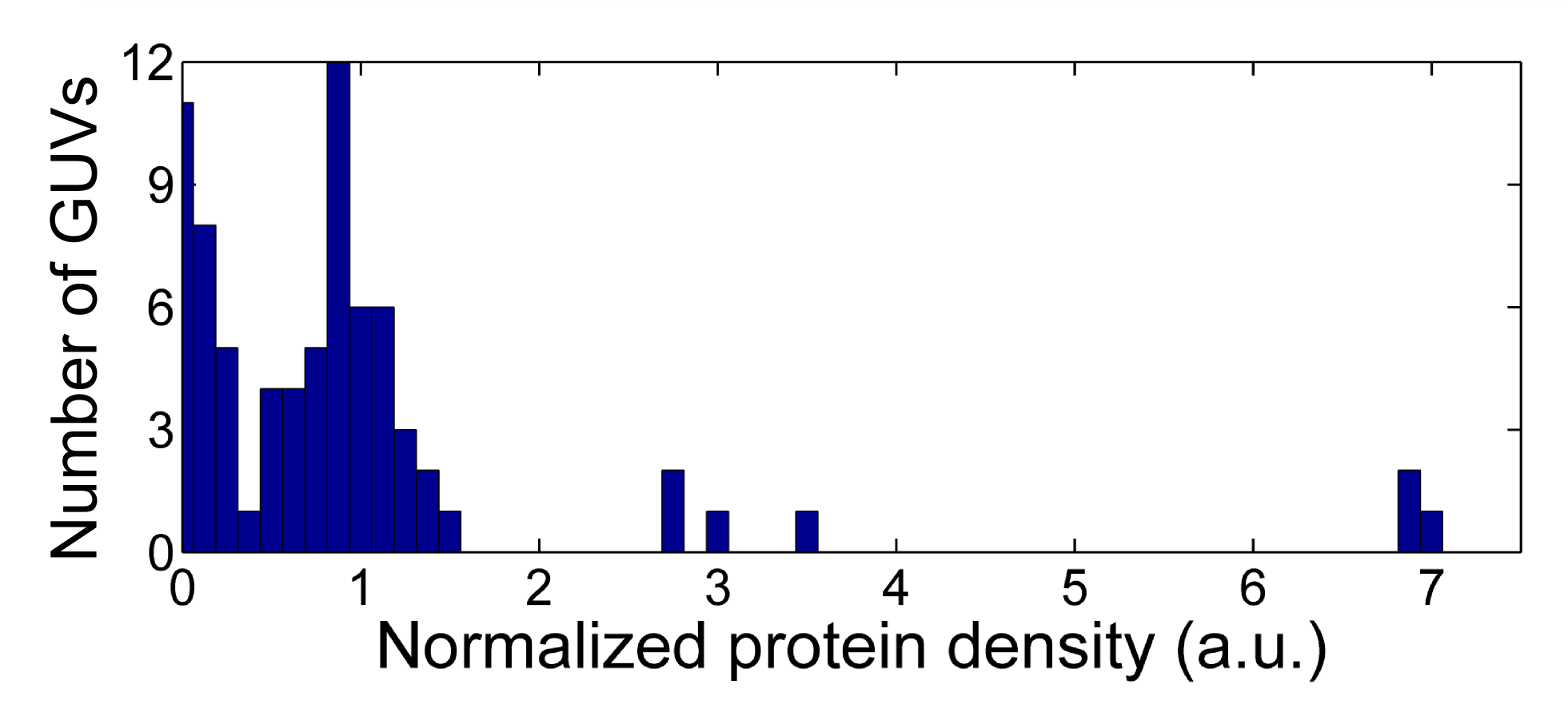

Supplement: Figure S10 — Variability of protein density in “high salt” GUVs is not due to multilamellarity. Protein density histogram (N = 75) of the population of high salt proteo-GUVs presented in Figure S8 (which were shown to be unilamellar via lipid fluorescence). Although the GUVs are unilamellar, the protein density still varies between GUVs with very high protein densities in a couple of GUVs. (TIF) [file pone.0025529.s010.tif]

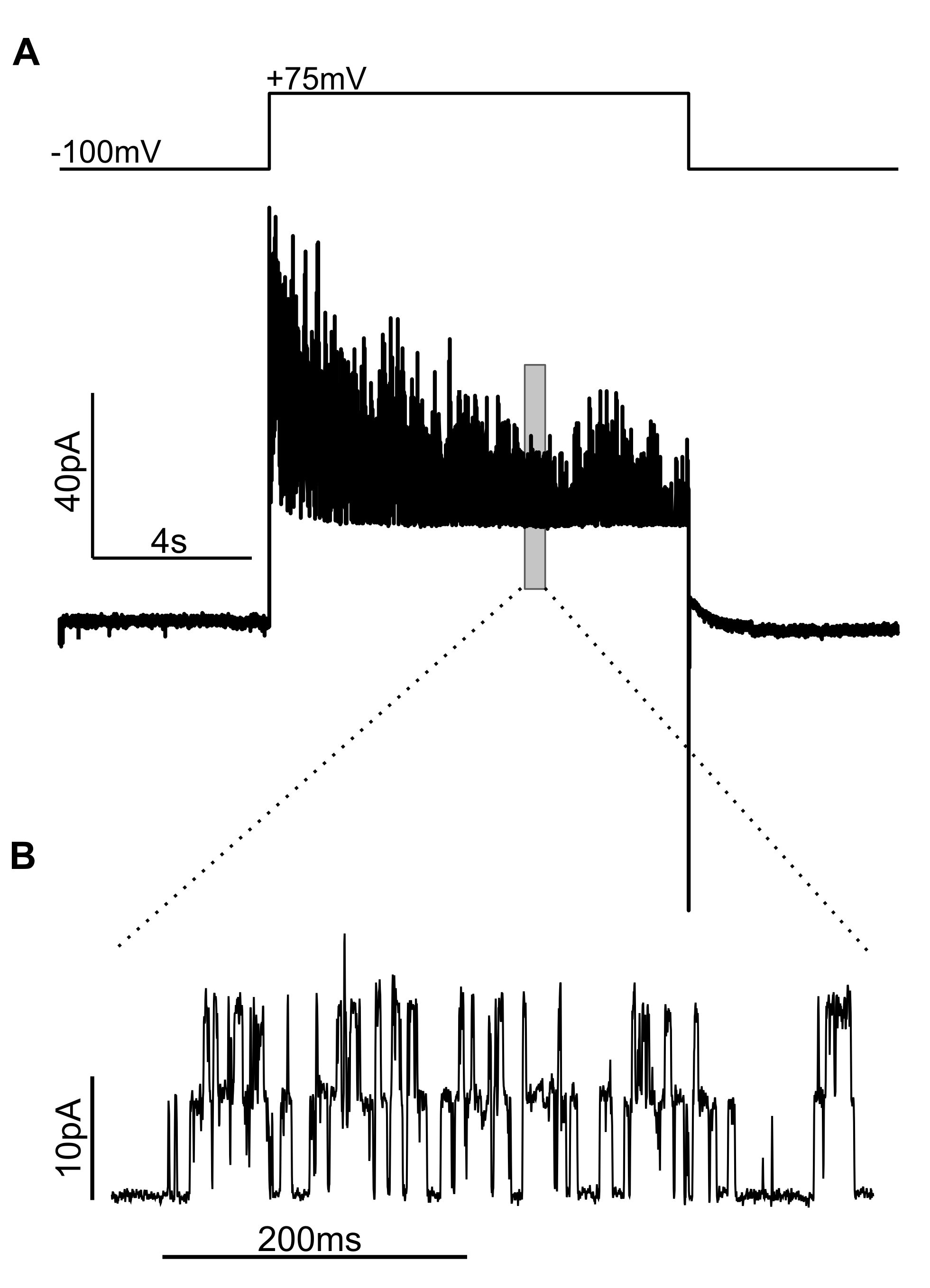

Supplement: Figure S11 — Activity of Membrane Patch from a GUV formed with low-salt buffer. A) GUV membrane patch current in response to an applied voltage step. Patch and bath solutions were both 100 mM KCl, 4 mM HEPES, pH 7.2, and the patch was formed from a EPC∶EPA (9∶1 by mole) GUV formed using low-salt buffer (5 mM) B) Section of the trace showing distinct jumps in conductance that are consistent with the opening and closing of individual channels. (TIF) [file pone.0025529.s011.tif]
