# Supplementary material for: Functional Reconstitution of a Voltage-Gated Potassium Channel in Giant Unilamellar Vesicles
Source: PLoS One. 2011 Oct 6;6(10):e25529. doi: 10.1371/journal.pone.0025529 (PMC3188570; doi:10.1371/journal.pone.0025529)
Supplement: Text S1 — KvAP Purification Protocol. Procedures for the expression and solubilization, purification, fluorescent labeling and reconstitution into proteoliposomes of KvAP. (PDF) [file pone.0025529.s012.pdf]

Supporting Text S1: KvAP Purification Protocol

Expression and Solubilization

The protocol for purifying KvAP was based upon the work of (Ruta et al. 2003). Pr. R. MacKinnon (Rockefeller University, NY) generously gave us the wild-type KvAP cDNA inserted into the pQE60 vector (Qiagen). This plasmid included the endogenous cysteine, C247 and a C-terminal hexa-histidine tag. For each purification, 100 µl of XL-1 Blue competent cells were transformed with the plasmid (100 ng) and incubated in 500 µl of SOC medium (Invitrogen) for 30 min at 37°C. The bacteria were then plated onto 6 LB/ampicillin agar plates and left overnight at 37°C. The following morning, all the colonies on the plates were transferred into 200 ml of LB containing 200 mg/L (0.6mM) ampicillin and this small culture was incubated for 1h at 37°C. The bacteria were then diluted into 6 baffled flasks (2L Nalgene Shake Flasks, Thermo Fisher Scientific) containing 1L of LB, 200 mg/L ampicillin and 10 mM BaCl2 and grown at 37°C under vigorous shaking (240 RPM).

After 4 to 5 hours, the culture reached an OD600 of 0.8-0.9 and protein expression was then induced by the addition of 0.4mM isopropyl-b-D-thiogalactopyranoside (IPTG, Euromedex). Following 4 hours of expression, cells were harvested via centrifugation (15 min, 8000 g) and the pellets stored overnight at 4°C. The following day, the cells (~30 g wet mass) were resuspended in 100 ml of a buffer containing 100 mM KCl, 50 mM Tris (pH8), 6 tablets of EDTA free proteinase inhibitors (Roche), 1 ml of 100 mM phenylmethylsulfonyl fluoride (PMSF, Fluka) and a small spatula of DNAse I (Roche diagnostics). Cells were broken by tip sonication on ice (8 cycles each consisting of 60 seconds of sonication (1 second on, 1 second off) followed by a pause, XL 2020 Sonicator, Misonix). 40mM n-Decyl-maltopyranoside (DM, Anagrade, Affymetrix, Maumee, OH) was then added to solubilize the cell membranes and the solution was gently agitated for three hours at room temperature. After solubilization, un-dissolved material (such as inclusion bodies) was removed by ultracentrifugation (30000g, 20 min, 10°C) and the resulting supernatant filtered (0.2 µm) in preparation for purification.

Purification

Initial purification of the protein was achieved via IMAC (Immobilized Metal ion Affinity Chromatography). Using an ÄKTAbasic system (GE Healthcare), a His-tag affinity column (1mL volume, HiTrap chelating column charged with Ni2+, GE Healthcare) was equilibrated with buffer A (5 mM DM, 100 mM KCl, and 20 mM Tris pH 8) and the lysate then loaded at a flow rate of 1ml/min. To remove non-specifically bound proteins, the column was washed first with ~5 ml of buffer A, and then with ~10 ml of buffer A + 15 mM imidazole. For each wash, absorbance at 280 nm was monitored and the wash was stopped when the absorbance returned to the baseline. KvAP was then eluted using buffer A + 400 mM imidazole. The most concentrated protein fractions were selected using the measured absorbance at 280nm. The typical yield after pooling these fractions was 5 mg of protein at a concentration of 2 mg/ml.

To remove the hexa-histidine tag, 2 units of thrombin (Roche diagnostics) were added and the solution left overnight at room temperature. The following morning, disulfide bonds were reduced by adding 1mM TCEP (Invitrogen). After allowing the reaction to proceed for 1 hour at room temperature, the protein was re-concentrated to ~10 mg/ml using a 10 kDa cutoff concentrator (Amicon) and filtered using a 0.2 µm Spin-X filter (Sigma). The final purification was achieved in 5 mM DM, 20 mM TRIS (pH 8), 100 mM KCl using a Superdex 200 10/300 GL (GE Healthcare) size exclusion column. Figure S1 shows a typical elution profile. After selecting the most concentrated fractions, the protein concentration was determined by measuring the absorbance with a Nanodrop UV-Vis absorption spectrometer (A280 = 37000 M-1cm-1). The final yield of the purification was typically around 3 mg protein.

Fluorescent labeling

Before reconstitution, the protein was fluorescently labeled with Alexa 488 maleimide (Invitrogen) at a final ratio of 0.2mg Alexa-488 per 1 mg of KvAP. After allowing the reaction to proceed for 2-4h at room temperature, the un-reacted free label was removed (along with the detergent) via dialysis during the reconstitution process. Fluorescence images of 12% SDS-PAGE gels (before staining with Coomassie Blue) showed that the channel was labeled as displayed in Figure S2. Proteins were typically labeled with 1-2 fluorophores per channel (see SupportingText S4).

Reconstitution in small liposomes

The protein was reconstituted in small liposomes following a protocol from (Schmidt, Jiang & MacKinnon 2006). EPC (L-_-phosphatidylcholine), EPA (L-_-phosphatidic acid) and DPhPC (diphytanoylphosphatidylcholine) were purchased from Avanti Polar Lipids (Alabaster, AL) and stock solutions were prepared by weighing lipids before dissolving them in chloroform.

Two lipid compositions were used for reconstitution: EPC:EPA (9:1 w:w) and DPhPC. Lipids in chloroform were dried under argon and any remaining solvent was then removed by placing the samples under a vacuum for 3-12h. The lipid was then suspended at 10mg/ml in a low-salt buffer (5 mM KCl and 1 mM HEPES pH 7.4). Small Unilamellar Vesicles (SUVs) were then formed by tip sonication (1sec on, 1sec off for 30 sec, ~5 times; XL 2020 Misonix). Note that solutions with DPhPC did not fully clarify after sonication.

The SUVs were then pre-solubilized by adding 10 mM decylmaltopyranoside (DM) for 30 min at RT. The protein was concentrated up to approximately 10 mg/ml in a 15 ml 30 kDa cutoff concentrator (Amicon) and was added to the pre-solubilized SUVs to achieve a protein:lipid ratio of 1:10 by mass. More DM was then added (final concentration of 17.5mM) and the resulting mixture was gently agitated for approximately 1h. Detergent was then removed by dialysis (12-14K tubing, Spectrum Laboratories, The Netherlands). The external buffer was changed every 6 to 12 h for 3 days, and after dialysis the resulting vesicles were aliquoted, flash-frozen in liquid nitrogen and stored at -80°C. These small proteoliposomes were used as stock for GUV preparation.

For fluorescently labeled KvAP, most of the free label was removed during dialysis but some Alexa-488 remained trapped inside vesicles. A possible alternative to avoid this problem would be to label the protein when bound to the His affinity column, or to pass the protein solution through a desalting column prior to the reconstitution. To confirm this free label did not influence GUV experiments, a control experiment was performed by preparing liposomes (fluorescent labeling, solubilization, dialysis and freezing steps) without protein. Although the resulting SUV solutions contained trapped Alexa-488, no fluorescence was detected in the membrane of GUVs prepared from these SUVs.

References

Ruta, V, Jiang, Y, Lee, A, Chen, J & MacKinnon, R 2003, 'Functional analysis of an archaebacterial voltage-dependent K+ channel', *Nature*, vol 422, no. 6928, pp. 180-185.

Schmidt, D, Jiang, Q & MacKinnon, R 2006, 'Phospholipids and the origin of cationic gating charges in voltage sensors', *Nature*, vol 444, no. December, pp. 775-779.
