# Supplementary material for: Functional Reconstitution of a Voltage-Gated Potassium Channel in Giant Unilamellar Vesicles
Source: PLoS One. 2011 Oct 6;6(10):e25529. doi: 10.1371/journal.pone.0025529 (PMC3188570; doi:10.1371/journal.pone.0025529)
Supplement: Text S2 — Proteo-GUV formation Protocol. Methods used to produce GUVs with reconstituted KvAP, GUVs containing co-existing liquid domains and pure lipid GUVs. (PDF) [file pone.0025529.s013.pdf]

# Supporting Text S2: Proteo-GUV formation Protocol

## Production of GUVs with reconstituted KvAP

The protocol to prepare GUVs containing KvAP was based upon that of (Girard et. al. 2004), with modifications to allow the use of physiological buffers (Meleard, Bagatolli & Pott 2009). Figure S3 illustrates the key steps of the electro-formation process. Droplets of a solution of SUVs containing the protein are deposited on wire electrodes. Partial evaporation of the solution leads to the formation of a lipid protein film through the fusion of SUVs. GUVs then form when this film is rehydrated under an applied AC electric field.

The protein-to-lipid ratio and lipid composition of the initial SUV solution was adjusted by combining a solution of the proteoliposomes (preparation described in Supporting Text S1) with other SUV solutions. For example, SUVs containing TR-DHPE were frequently added (final concentration of up to 0.5% by mole) to fluorescently label the membrane. The SUVs solution was homogenized by vortexing for approximately 1 min. Sonication of the SUV solution did not appear to produce GUVs with more uniform fluorescence. Because the salt concentration increases significantly during the partial dehydration step, SUVs were always prepared in a“low-salt” buffer (5mM KCl, 1mM HEPES pH7.4). In addition, 2 to 10 mM of trehalose (generously given by Prof. Lorenzo Cordone, Palermo, Italy) was added to the SUV solution to further protect the protein during the partial dehydration step (Crowe, Reid & Crowe 1996). The final lipid concentration in the SUV mixture was typically ~3 mg/ml.

As shown in Figure S4, the electro-formation chamber was made from a Teflon block containing three wells (diameter ~10mm). The electrodes consisted of two parallel platinum wires (0.5mm or 0.8 mm in diameter, Goodfellow, UK) separated by a edge-to-edge distance of 2 mm. Tests showed that GUVs could also be formed using titanium wires in place of the platinum wires. Before each GUV growth, the wires were typically cleaned by rubbing with Kimwipe tissues (Kimberly-Clark), washes with water, ethanol and chloroform and bath sonication in these same solvents. Electrolysis of a 1M NaCl solution (V=7V RMS; f=1Hz) for 60 seconds was found to clean the wires very effectively and helped ensure a good yield of vesicles afterwards.

To form the protein-lipid film on the platinum wires, the proteoliposome mixture (5 μl of solution per 8 cm of platinum wire) was applied using a 10μl glass syringe (Hamilton, Bonaduz,Switzerland). Small droplets of the SUV solution were spread along the wire while taking care not to touch droplets to each other (to avoid merging droplets). The droplets were left to dry under room atmosphere for approximately 30 minutes: no liquid could be detected by eye after approximately 5 to 10 minutes and evaporation was typically allowed to continue for an extra 20 minutes. Insufficient dehydration of the SUV solution resulted in GUVs with non uniform membranes or even a dispersion of SUVs in solution during the rehydration. The use of a SUVsolution with a high osmolarity (e.g. 100mM KCl) further exacerbated such problems. In principle, excessive dehydration could damage the protein, so the shortest period of dehydration consistent with a good GUV yield was used.

After the deposition of the SUVs, the chamber bottom was sealed with a glass coverslip held in place by grease (Silicon Paste P4, Wacker). The wells were then carefully filled with GUV hydration buffer and the top of the chamber was then sealed with a second glass coverslip.

To apply a sinusoidal voltage, a signal generator (TG315 or TG215, TTi, or GFG 2004, Iso-tech) was connected to the two electrodes by alligator clips and the voltage across the two electrodes measured with a multi-meter. The voltage applied during GUV growth was chosen according to the GUV rehydration buffer. To grow GUVs in a "physiological" buffer of 200mM sucrose, 100mM KCl, 10mM Hepes pH 7.4, a voltage of 0.35V RMS (Root Mean Square) and 500Hz was applied overnight. For GUVs grown in "low-salt" buffer (5mM KCl, 1mM HEPES (pH7.4), 400mM sucrose) a 10 Hz voltage was used. The voltage was slowly stepped up to the maximal value of 0.7 V RMS over 30 minutes before continuing the growth for approximately 2 hours. Because the electric field could potentially affect protein conformation, efforts were made to use the lowest voltage/shortest time that gave a useful GUV yield.

The growth of GUVs on the electrodes could be followed via phase-contrast microscopy as shown in Figure S5. Note that GUVs typically formed in bunches along the wires. Curiously, when using DPhPC SUVs a surprising number of extremely large GUVs formed at the ends of each well where the wires made contact with the Teflon block.

As PEG lipids are thought to help produce defect-free GUVs (Streicher et al. 2009), growth was tested with up to 1% by mole of PEG-DOPE in the initial SUV mix. Similarly, to try to exclude the possibility of divalent ions cross-linking the dehydrated membranes stacks, GUV growth was also tested in the presence of 2 mM of EDTA. However, the presence of trehalose, EDTA or PEG lipids did not significantly affect GUV growth and the effects of trehalose, EDTA or PEG lipids on channel function were not studied.

## Preparation of GUVs containing co-existing liquid domains

To prepare SUVs containing DPPC:Cholesterol (1:1 by mole), lipids in chloroform were mixed, dried at 50°C for several minutes, and then kept under vacuum overnight. The lipid film was then rehydrated in a solution of 2 mM trehalose and sonicated as described in Supporting TextS1. SUVs containing DPhPC and TR-DHPE were prepared in the same way. To prepare vesicles with domains, DPhPC proteo-SUVs were mixed with these DPPC:Cholesterol SUVs to achieve a final lipid molar composition of 6:2:2 (DPhPC:DPPC:Cholesterol) plus 0.5% TR-DHPE. This composition has been reported to produce GUVs with co-existing liquid domains at room temperature (Veatch, Gawrisch & Keller 2006). 10 mM trehalose was then added to the SUVs mixture. To try to prevent de-mixing of the lipids during the GUV growth, the SUVs were dried at 50°C and rehydrated at 50°C in low-salt buffer (5mM KCl, 400mM sucrose, 2mM HEPES (pH 7.3)). During rehydration, a voltage of 0.5V RMS (10Hz) was applied for 30 min. For imaging, the resulting GUVs were then transferred into a buffer of 200mM glucose, 100mM NaCl, and 10mM Hepes (pH 7.3) at room temperature.

## Production of pure lipid GUVs

GUVs containing only lipids were prepared using a standard electro-formation protocol (Angelova et al. 1992). The stock solution for unilamellarity measurements was prepared by adding TR-DHPE (0.5% by mole) to a solution of EPC in chloroform (0.5mg/ml). For membrane fluorescence quantification, Bodipy-HPC was added to a solution of EPC in chloroform to form stock solutions with concentrations between 0.008% and 0.5% by mole.

25µl of the lipid solutions (0.5 mg/ml) was spread with a Hamilton syringe onto two ITO slides (Präzisions Glas & Optik, Iserlohn, Germany). Residual solvent was removed by placing the slides under vacuum for 2 hours to overnight. The slides were then assembled into a chamber (1mm thickness) using sigillum wax (Vitrex Medical, Herlev, Denmark), a 200mM sucrose buffer added, and an alternating voltage of 1.1 V RMS and 10 Hz applied for approximately 1 hour.

## References

Angelova MI, Soleau S, Meleard P, Faucon F, Bothorel P (1992) Preparation of giant vesicles by external AC electric fields. Kinetics and applications. In Helm, C., Losche, M., Mohwaldt, H., eds. *Trends in Colloid and Interface Science VI* **89**. Springer 127-131.

Crowe, LM, Reid, DS & Crowe, JH 1996, 'Is trehalose special for preserving dry biomaterials?', *Biophysical Journal*, vol 71, no. 4, pp. 2087-2093,

Girard, P, Pecreaux, J, Lenoir, G, Falson, P, Rigaud, J-L & Bassereau, P 2004, 'A new method for the reconstitution of membrane proteins into giant unilamellar vesicles.', *Biophysical journal*, vol 87, no. 1, pp. 419-429.

Streicher, P, Nassoy, P, Barmann, M, Dif, A, Marchi-Artzner, V, Brochard-Wyart, F, Spatz, J & Bassereau, P 2009, 'Integrin reconstituted in GUVs: A biomimetic system to study initial steps of cell spreading', *Biochimica et Biophysica Acta*, vol 1788, pp. 2291-2300.

Veatch, SL, Gawrisch, K & Keller, SL 2006, 'Closed-loop miscibility gap and quantitative tie-lines in ternary membranes containing diphytanoyl PC.', *Biophysical journal*, vol 90, no. 12, pp. 4428-36.
