# Supplementary material for: Functional Reconstitution of a Voltage-Gated Potassium Channel in Giant Unilamellar Vesicles
Source: PLoS One. 2011 Oct 6;6(10):e25529. doi: 10.1371/journal.pone.0025529 (PMC3188570; doi:10.1371/journal.pone.0025529)
Supplement: Text S3 — Characterization of the Proteo-GUVs. Description of methods used to characterized vesicle unilamellarity, size distribution and protein incorporation. (PDF) [file pone.0025529.s014.pdf]

Supporting Text S3: Characterization of the Proteo-GUVs

Vesicle Unilamellarity

To distinguish between unilamellar and multilamellar objects, proteo-GUVs were prepared containing the red fluorescent lipid, TR-DHPE. Membrane fluorescence should then be proportional to the number of bilayers in the vesicle. To establish the fluorescence signal of a single membrane, reference GUVs were prepared using a classical electro-formation protocol known to produce a high yield of unilamellar vesicles (Mathivet, Cribier & Devaux 1996).

Proteo-GUVs were prepared from a SUV mixture with a molar composition of 89% EPC, 9.5%EPA, 0.5% TR-DHPE and 1% PEG-DOPE following the protocol described in Supporting TextS2. Reference GUVs (99.5% EPC, 0.5% TR-DHPE) were prepared using the protocol described in last section of Supporting Text S2. The same stock solution of TR-DHPE was used for both types of GUV. After preparation, proteo-GUVs were collected using a 200µl pipette tip cut so the end formed a V shape. The pipette tip was scraped along the wires while aspirating, thereby collecting almost all the lipid material on the wire. This especially aggressive harvesting method was used in an effort to collect everything produced during the growth.

The GUV solution was transferred into an observation chamber containing an iso-osmolar solution and the GUVs were imaged via confocal microscopy. Figure S7 shows a typical field of view. Approximately half the objects transferred into the observation chamber were spherical and surrounded by a membrane that presented a single line (to within the limits of the optical resolution of the microscope). These objects thus appeared to be GUVs. To determine if the membrane fluorescence intensity of these "apparent GUVs" was consistent with a single lipid bilayer, confocal images of vesicle equatorial plane were analysed as described in Supporting Text S4. As shown in Figure S8, the membranes of ITO GUVs (notionally unilamellar), proteo-GUVs prepared with low salt and proteo-GUVs prepared with 100mM salt all have very similar mean lipid fluorescence (2000 ±260 (a.u.)). Indeed, of the 142 protein-containing vesicles analysed, only 2 were obviously bi-lamellar (2 vesicles with an intensity of ~4500 for low salt preparation). Thus, the intensity of lipid fluorescence from "apparent GUVs" containing protein is entirely consistent with a membrane consisting of a single bilayer.

Size Distribution

The size distribution of proteo-GUVs was evaluated using confocal images of the proteo-GUVs. Each GUV was analysed as described in Supporting Text S4 and the GUV diameter determined as the width of the GUV contour. As shown in Figure S6, proteo-GUVs with EPC/EPA lipids were approximately 10 µm in diameter. Note that increasing voltage and electroformation duration seemed to increase GUV size. Also DPhPC appeared to form larger GUVs.

Protein Incorporation

A detailed description of the measurement of protein density in GUVs is given in Supporting Text S4. This section summarizes the apparent dependence of protein incorporation on the GUV growth protocol.

In general, GUVs prepared with low-salt buffer had a narrower distribution of protein densities than GUVs prepared with "physiological" salt buffer. Figure S9 presents protein density histograms for two extreme cases. The very homogenous distribution shown in the upper histogram corresponds to a batch of proteo-GUVs prepared using electroformation in low salt buffer. The extremely broad distribution shown in the lower histogram corresponds to a batch of GUVs prepared via a long electroformation in "physiological" salt buffer. For this batch of GUVs, many vesicles contained nearly no protein and a few contained a high protein density. It is important to note that even though the protein density was greatly different between GUVs, the protein was still homogeneously distributed within each GUV. To exclude the possibility that vesicles highly enriched in protein were in fact multilamellar, protein density was measured for GUVs containing TR-DHPE grown using the "physiological" salt buffer. For these GUVs, lipid fluorescence could be used to detect multilamellar objects. As shown Figure S10, the wide protein density distribution is not due to multilamellar objects.

Reference

Mathivet, L, Cribier, S & Devaux, P 1996, 'Shape change and physical properties of giant phospholipid vesicles prepared in the presence of an AC electric field', *Biophysical Journal*, vol 70, no. 3, pp. 1112-1121.
