# Supplementary material for: Functional Reconstitution of a Voltage-Gated Potassium Channel in Giant Unilamellar Vesicles
Source: PLoS One. 2011 Oct 6;6(10):e25529. doi: 10.1371/journal.pone.0025529 (PMC3188570; doi:10.1371/journal.pone.0025529)
Supplement: Text S4 — Measurement of Protein Density. Explanation of the fluorescence calibration method, quantification of membrane fluorescence intensity, area of the GUV membrane in the confocal volume and fluorophores per protein. (PDF) [file pone.0025529.s015.pdf]

Supporting Text S4: Measurement of Protein Density

The density of fluorescently labeled protein in GUVs was measured via confocal microscopy. In principle, the molecular concentration per µm^2, D, and the fluorescence intensity, I, should be related by the equation,

$$D = \frac{I}{N_f \times a \times I_0}$$

where N_f is the number of fluorophores per molecule, a is the effective membrane area in the confocal volume, and I_0 is the intensity per fluorophore. Thus, in addition to measuring the fluorescence intensity, I, one must also determine the product of N_f, a and I_0. The number of fluorophores per molecule, N_f, can be directly determined via absorbance spectroscopy. In contrast, a and I_0 depend upon many factors including the orientation of the membrane (horizontal, vertical, etc.), the nature and environment of the fluorophore, the intensity of the fluorescence excitation source and the efficiency of the detector. Recently, (Galush, Nye & Groves 2008) introduced an elegant method to determine the product, $a*I_0$, through the use of a reference fluorophore (e.g. fluorescent lipid) that can be incorporated into membranes at known density. Adapting this approach to confocal images of GUV membranes, the calibration is performed by measuring the effects of:

1. Geometry:
   Measurements of the fluorescence intensity, I_{ref}, of similar GUVs containing a reference fluorophore (lipid with 1 fluorophore per lipid) at known density $D_{ref,}$ are used to determine the product,

   $$M_{ref} = a \times I_{0,ref} = \frac{I_{ref}}{D_{ref}}$$

2. Relative fluorescence intensity:
   Measurements of fluorescence from bulk solutions (SUVs or detergent micelles) are used to compare the intensity of the protein fluorescence relative to the reference fluorophore,

   $$F = \frac{I_{0,protein}}{I_{0,ref}}$$

   Combining these two measurements then gives,

$$D = \frac{I}{N_f \times (a \times I_{0,ref}) \times \left(\frac{I_{0,protein}}{I_{0,ref}}\right)} = \frac{I}{N_f \times M_{ref} \times F}$$

The following sections describe the measurement of

- Membrane fluorescence
- Effect of geometry
- Fluorescence yield
- Fluorophores per protein

and their final application to determine the density of proteins in GUVs.

Measuring Membrane Fluorescence

GUV membrane fluorescence was measured by imaging the vesicle equatorial plane with a Nikon TE 2000 C1 confocal microscope using a 100x Nikon Plan-Fluor oil objective (N.A.=1.3). GUV images were analyzed using a Matlab (Mathworks, MA) program. For each GUV, the membrane contour was fitted to the form of an ellipse and the intensity profile was then determined by averaging along the contour (Figure 3A). In cases where part of the contour could not be used (e.g. when two GUVs were too close together), the average was taken on the section of the contour where the membrane of the GUV could be clearly isolated. The fluorescence intensity of the membrane, I, was defined to be the maximum of the intensity profile minus the background fluorescence level.

Several factors were important to accurately determine the fluorescence intensities.

Firstly, all relevant confocal parameters (PMT gain and offset, pixel dwell, pixel size, laser power, pinhole size, and position of GUV in the confocal field) were either held constant, or the effect of any changes was corrected for. For example, a calibration was performed to allow comparison of intensities (I_0, I_1) obtained with different PMT gains ($Gain_0$, $Gain_1$) using the equation, $I_1 = I_0 \times exp(\beta \times (Gain_1 - Gain_0))$, where β describes the scaling of the PMT gain ($\beta \sim 0.068$). To obtain reliable results, confocal parameters were adjusted so that the fluorescent signal strength was significantly larger than the PMT noise but did not saturate the detector.

To avoid bleaching of the fluorophores (Alexa 488, Bodipy-HPC), care was taken to avoid exposure of the GUVs to bright light before the measurement. For typical imaging conditions (pixel size of approximately 100 nm and a dwell time of 1.68 μs), the fluorescence intensity diminished by less than a few percent per image. As only one image was taken for fluorescence measurements, the bleaching was negligible.

Clearly, the measured fluorescence intensity depended upon excitation intensity and detector efficiency. Changes in fluorescence intensity were observed over a time-scale of weeks to months, probably due to misalignment or aging of the laser. These variations were accounted for by regularly measuring the intensity of a solution of known fluorophore concentration (e.g. 10μM of Alexa 488).

Effect of geometry

As described above, fluorescence intensity measurements were calibrated using GUVs containing known concentrations of the green fluorescent lipid, Bodipy-HPC.

GUVs were prepared with Bodipy-HPC at 0.008% to 0.5% by mole (see Supporting Text S2). The relative concentrations of Bodipy-HPC solutions were determined by measuring theabsorbance at 509 nm (ε ≈ 70000 M-1cm-1; Invitrogen) of a sample that had been resuspended at a concentration of 0.02mg/ml in a solution of 100mM NaCl, 200mM glucose, 10mM Hepes (pH7.4), and 50 mM of DM. EPC and EPA stock solutions were prepared by weighing the lipid in powder form before dissolving them in chloroform. The number of

fluorophores per μm^2 was then calculated by assuming a lipid head size of 0.7 ± 0.1 nm^2 (corresponding to the size of EggPC lipids (Nagle & Tristram-Nagle 2000)). Accounting for the two leaflets, the density of lipids is then $\frac{2\times10^6}{0.7} = (2.9 \pm 0.4) \times 10^6$ lipids per square micron. This range of Bodipy-HPC concentrations was large enough to effectively test the linearity of the detector over the intensity range studied while still avoiding fluorophore saturation (Galush, Nye & Groves 2008).

For each fluorophore density, approximately 10 vesicles were analyzed. As shown in Figure 3B of the manuscript, the fluorescence intensity depended linearly on the fluorophore concentration and the slope of the plot was equal to $M_{ref} = a \times I_{0,ref}$. To expand the range of the calibration,measurements were performed at several PMT gain settings.

Fluorescence yield

To compare the relative fluorescence intensity of Bodipy-HPC and KvAP-Alexa 488, thefluorescence from homogenous solutions of these two fluorophores were measured with theconfocal microscope.

SUVs containing Bodipy-HPC or the fluorescent protein were prepared as described in Supporting Text S1. Several solutions with different fluorophore concentrations (1 to 20 μM) were then obtained by diluting the SUVs in the external buffer solution (200mM glucose,150mM NaCl, 10mM HEPES at pH 7.3). The absorbance of the solutions containing DM was measured with a Nanodrop (Thermo Scientific) to determine fluorophore concentration (A_{509} ≈70000 M^{-1} cm^{-1} for Bodipy-HPC and A_{494}= 71000 $M^{-1}cm^{-1}$; Invitrogen). Each solution was then loaded into a chamber on the microscope and images taken at 3 different places in the chamber. To match the GUV imaging conditions, the objective was focused at a height of 10 ns above the coverslip. The mean intensity of the fluorescence was then measured in a region near the optical axis of the microscope where the intensity was most uniform.

Although the SUVs in solution should have been much smaller than the optical resolution of the microscope, bright saturated spots in the images suggested the presence of lipid aggregates. As these spots saturated the PMT, they could have introduced a non-linearity into the measurements and attempts were made to eliminate them. Additional sonication of the solutions produced more homogenous images, but also reduced the overall fluorescence suggesting that sonication can damage fluorophores. Dissolving the SUVs in detergent (50 mM DM) also produced a homogeneous solution, but the presence of detergent may affect the fluorophore environment and yield. However, the fluorescence intensity of SUVs and SUVs dispersed in detergent were quite similar, suggesting that aggregates did not strongly influence the measurements.

For both Bodipy-HPC and KvAP-Alexa488, the fluorescence intensity was proportional to fluorophore concentration. The ratio of fluorescence intensity per fluorophore was found to be:

$$F = \frac{I_{0,Alexa\_KvAP\_bulk}}{I_{0,Bodiopy-HPC\_bulk}} = 0.94 \pm 0.10$$

Note that this measurement is for an isotropic solution. In contrast, in GUVs the fluorophores can have a net orientation relative to the bilayer normal. If the reference fluorophore and protein fluorophore have different net orientations, the fluorescence yield ratio in GUVs may be altered by a polarization factor.

Fluorophores per protein

The number of fluorophores per protein was measured via absorbance spectroscopy. Samples were prepared by solubilizing a solution of proteo-SUVs (at approximately 1mg/ml of protein and 10 mg/ml of lipids) in 50mM DM followed by dialysis to remove any free Alexa-488 that may have been trapped inside the SUVs. The concentrations of the protein and of Alexa-488 were then measured with a Nanodrop spectrophotometer ($A_{494}^{Alexa} \approx 71000\ M^{-1}cm^{-1}$, $A_{280}^{KvAP} = 37360\ M^{-1}\ cm^{-1}$). The labeling ratio was N_f = 1.7±1.0 per channel (0.42 ± 0.24 per KvAP monomer). The uncertainty in N_f was dominated by the difficulty to measure protein concentration in the presence of a high absorption background caused by the presence of lipids and detergent. This difficulty could in principle be avoided by measuring the number offluorophores per protein prior to reconstituting the protein into SUVs.

Protein density in GUVs

GUVs were prepared using the low salt protocol (Supporting Text S2). Briefly, for this experiment, proteo-SUVs were diluted in 2 mM trehalose down to 2 mg/ml. The rehydration buffer was sucrose 400 mM, KCl 5 mM, Hepes 1 mM pH 7,3, EDTA 2 mM. For the data shown in Figure 4, the SUVs had a protein:lipid mass ratio of 10.5 ± 2.5. For a mean lipid molecular mass (770×0.9+697×0.1=763 g/mol for our EPC:EPA 9:1 mix) and channel mass of 4×30860=123440 Da, this corresponds to $(1.8 \pm 0.4)\times 10^3$ lipids per protein. The protein densityin the SUVs should then be (1.6 ± 0.4)×103 proteins per square micron.

The fluorescence intensity of GUVs was measured as described previously. Interestingly, GUVs containing a high concentration of protein did not sediment rapidly and had low contrast when imaged in DIC. These observations suggest that sucrose/glucose was able to exchange across the membrane, which might result from a failure to completely eliminate bacterial porins during the purification (Accardi et al. 2004), or a bacterial contamination in the proteo-SUVs. However, any contamination by porins must be minor as they were not observed during patch-clamp recordings of GUVs. To allow as many GUVs as possible to sediment to the bottom, measurements were only started one hour after transferring GUVs to the observation chamber.

For each vesicle, the protein density was calculated using,

$$D = \frac{I}{N_f \times M_{ref} \times F}$$

and the results for N=72 vesicles are shown in Figure 4. The mean protein density in GUVs was $(1.0 \pm 0.7)\times 10^3$ per square micron, which was similar to the protein density for the SUVs from which they were formed.

To summarize, protein density in GUVs was measured using the fluorescence of labeled KvAP. A calibration using vesicles containing a controlled amount of a reference fluorophore was used to relate membrane intensity to fluorophore density. The fluorescence yield ratio between the reference fluorophore and protein was then used to directly relate membrane fluorescence intensity to protein density.

References

Accardi, A, Kolmakova-Partensky, L, Williams, C & Miller, C 2004, 'Ionic Currents Mediated by a Prokaryotic Homologue of CLC Cl-Channels', *The Journal of General Physiology*, vol 123, no. 2, pp. 109-119,

Galush, WJ, Nye, JA & Groves, JT 2008, 'Quantitative fluorescence microscopy using supported lipid bilayer standards.', *Biophysical Journal*, vol 95, no. 5, pp. 2512-9.

Nagle, JF & Tristram-Nagle, S 2000, 'Structure of lipid bilayers', *Biochim Biophys Acta*, vol 1469, no. 3, pp. 159-95.
